# Global spatially explicit CO_2_ emission metrics for forest bioenergy

**DOI:** 10.1038/srep20186

**Published:** 2016-02-02

**Authors:** Francesco Cherubini, Mark Huijbregts, Georg Kindermann, Rosalie Van Zelm, Marijn Van Der Velde, Konstantin Stadler, Anders Hammer Strømman

**Affiliations:** 1 Industrial Ecology Programme, Department of Energy and Process Engineering, Norwegian University of Science and Technology (NTNU), Trondheim, Norway; 2 Department of Environmental Science, Institute for Water and Wetland Research, Radboud University, Nijmegen, The Netherlands; 3 Dutch Environmental Assessment Agency, Bilthoven, The Netherlands; 4 Ecosystems Services and Management Program (ESM), International Institute for Applied Systems Analysis (IIASA), Laxenburg, Austria; 5 European Commission, Joint Research Centre (JRC), Ispra, Italy

## Abstract

Emission metrics aggregate climate impacts of greenhouse gases to common units such as CO_2_-equivalents (CO_2_-eq.). Examples include the global warming potential (GWP), the global temperature change potential (GTP) and the absolute sustained emission temperature (aSET). Despite the importance of biomass as a primary energy supplier in existing and future scenarios, emission metrics for CO_2_ from forest bioenergy are only available on a case-specific basis. Here, we produce global spatially explicit emission metrics for CO_2_ emissions from forest bioenergy and illustrate their applications to global emissions in 2015 and until 2100 under the RCP8.5 scenario. We obtain global average values of 0.49 ± 0.03 kgCO_2_-eq. kgCO_2_^−1^ (mean ± standard deviation) for GWP, 0.05 ± 0.05 kgCO_2_-eq. kgCO_2_^−1^ for GTP, and 2.14·10^−14^ ± 0.11·10^−14^ °C (kg yr^−1^)^−1^ for aSET. We explore metric dependencies on temperature, precipitation, biomass turnover times and extraction rates of forest residues. We find relatively high emission metrics with low precipitation, long rotation times and low residue extraction rates. Our results provide a basis for assessing CO_2_ emissions from forest bioenergy under different indicators and across various spatial and temporal scales.

Bioenergy is currently the most important renewable energy option in the global primary energy mix^1^, and its contribution is expected to further increase in the near future^2^,^3^. Many studies find that a stabilization of the global temperature rise requires phasing out fossil fuels while increasingly relying on terrestrial ecosystems to supply energy and materials^2^,^4^. Close to 1.2 billion hectares of forests are today actively managed for the production of wooden products, with wood fuel accounting for about half of the reported wood removals^5^. Clear-cut logging is the main cause of human-induced stand-replacing forest disturbances, and it is likely to become more frequent in the future according to the predicted growing biomass for energy demands^4^,^5^,^6^.

The climate change impacts of forest bioenergy systems are frequently assessed in Life-Cycle Assessment (LCA) studies^7^, carbon footprint estimates of countries and products^8^, and international policy directives and frameworks^9^, under a default “carbon neutrality” assumption that ignores the temporal asymmetry between fast emissions from biomass combustion and slow CO_2_ uptake by vegetation re-growth. Harvesting forests alters the net exchange of CO_2_ between the land and atmosphere, with consequences for the CO_2_ atmospheric concentration and climate system^10^,^11^. Post-harvest forest stands are usually a source of carbon for some years after disturbance because CO_2_ emissions from heterotrophic respiration (R_h_) exceed carbon sequestration in new trees via net primary productivity (NPP)^11^,^12^,^13^. Once residues have decomposed and NPP increases, the net ecosystem exchange (NEE, NEE = Rh – NPP) becomes negative, and the forest ecosystem acts as a net carbon sink. The transition from carbon source to carbon sink usually occurs within the first two or three decades following stand replacement^12^, and largely depends on the amount and decay rate of post-harvest forest residues remaining in the forest to decompose^14^.

Human-induced forest disturbances typically involve within-class land conversion (e.g., forest land remaining forest land) that can affect the global climate as much as conversion to a different type of land cover class^11^,^15^. As synthesized by the 5^th^ IPCC Assessment Report^16^,^17^, bioenergy systems cause a temporary climate impact even when the net CO_2_ fluxes sum to zero over time. Such impacts can be assessed at different points of the carbon-climate cause-effect chain^16^, from the estimation of a carbon payback time based on a mass balance of CO_2_ fluxes^18^,^19^ to radiative forcing and temperature changes^20^,^21^. Site-specific emission metrics^7^,^22^,^23^,^24^ can be used to aggregate the climate impacts from bioenergy CO_2_ emissions with those from other GHGs in terms of CO_2_-equivalents. Emission metrics are simplified measures of the climate system response to GHG emissions and are based on outcomes from complex models^16^. These metrics are formulated to be transparent and easily applied by non-specialists without expert input at the point of use^25^ and are thus widely utilized. Applications span from LCAs and carbon footprints to international agreements, such as the Kyoto protocol, and legislative frameworks^16^,^25^,^26^,^27^. Metrics are typically classified according to three criteria^26^: i) emission type, i.e., for a single pulse or emission scenarios; ii) indicator, i.e., radiative forcing or temperature change; iii) time dimension, i.e., the indicator is used in its instantaneous or time-integrated form, with absolute or normalized (e.g., with CO_2_ taken as the reference gas, hence CO_2_-equivalents) values taken at a specific time horizon (TH). Different emissions have different climate system responses, and a metric that establishes equivalence with regard to one effect cannot guarantee equivalence with regard to other effects. Various metrics are therefore available, and the choice of a metric should depend on the aspect of climate change that is identified as the most important in a particular application^16^. In this work, we focus on three metrics, GWP^16^, GTP^16^,^27^, and aSET^28^, among a range of others available in the climate science literature^16^,^25^,^26^,^27^. The GWP is defined as the time-integrated radiative forcing of a pulse emission until an arbitrary TH divided by an equivalent integration for CO_2_^16^. Despite its name, the GWP does not lead to equivalence of emissions on the basis of impacts on surface temperature^29^. The Global Temperature change Potential (GTP) was proposed as an alternative^27^. It is defined as the change in global mean surface temperature at the chosen TH following an emission pulse, again relative to CO_2_. GWP and GTP thus differ in the indicator (radiative forcing vs. temperature change) and time dimension (time-integrated vs. instantaneous). By contrast, aSET is an absolute metric that refers to the contribution to a global mean temperature peak (ΔT_peak_), with no time dimensions. This metric is used for emission scenarios of shorter-lived climate forcers, with which bioenergy CO_2_ emissions can be grouped because they cause ΔT_peak_ values dependent on emission rates rather than cumulative emissions^20^.

Emission metrics for forest bioenergy are currently available for only a handful of specific locations^18^,^19^,^22^,^23^,^30^. The quantification of these metrics requires knowledge and processing of a wide spectrum of modelled or observed local climate and forest conditions, and a spatially explicit analysis of emission metrics with global forest coverage is lacking. Here, we develop and apply spatially-explicit climate change emission metrics for CO_2_ emissions from forest bioenergy at a global scale. Emission metrics are computed at 0.25° spatial resolution by coupling a global forest carbon model (G4M)^31^,^32^ with the heterotrophic respiration model YASSO07^33^ and the climate impact protocol used by the IPCC for emission metrics and temperature responses^16^,^29^ (see Methods). Local climate variables^34^ and forest structure information (such as mean annual increments, turnover times, biomass stocks, etc.; see Supplementary Figure S1) are used to simulate post-harvest NEE fluxes per grid cell under different extraction rates of forest residues. GWP is evaluated at a TH of 100 years, which is the most common TH used in LCAs or emission reporting schemes such as the Kyoto Protocol. GTP is computed for a TH of 85 years to quantify the impact of emissions in 2015 on the global average surface temperature in 2100, which is the internationally recognized target year by which global warming should be maintained at less than 2 °C above pre-industrial levels^2^. We plot the emission metrics against climate variables and turnover times and present the aggregated findings at the grid, country and continental levels. We demonstrate an application of these spatially explicit metrics through the characterization of the global CO_2_ emissions from bioenergy in 2015 and until 2100 under the Representative Concentration Pathway (RCP) 8.5 scenario^35^, for which gridded wood fuel combustion flows are available^36^.

## Results

### Spatially explicit emission metrics for forest bioenergy

**Figure 1:**
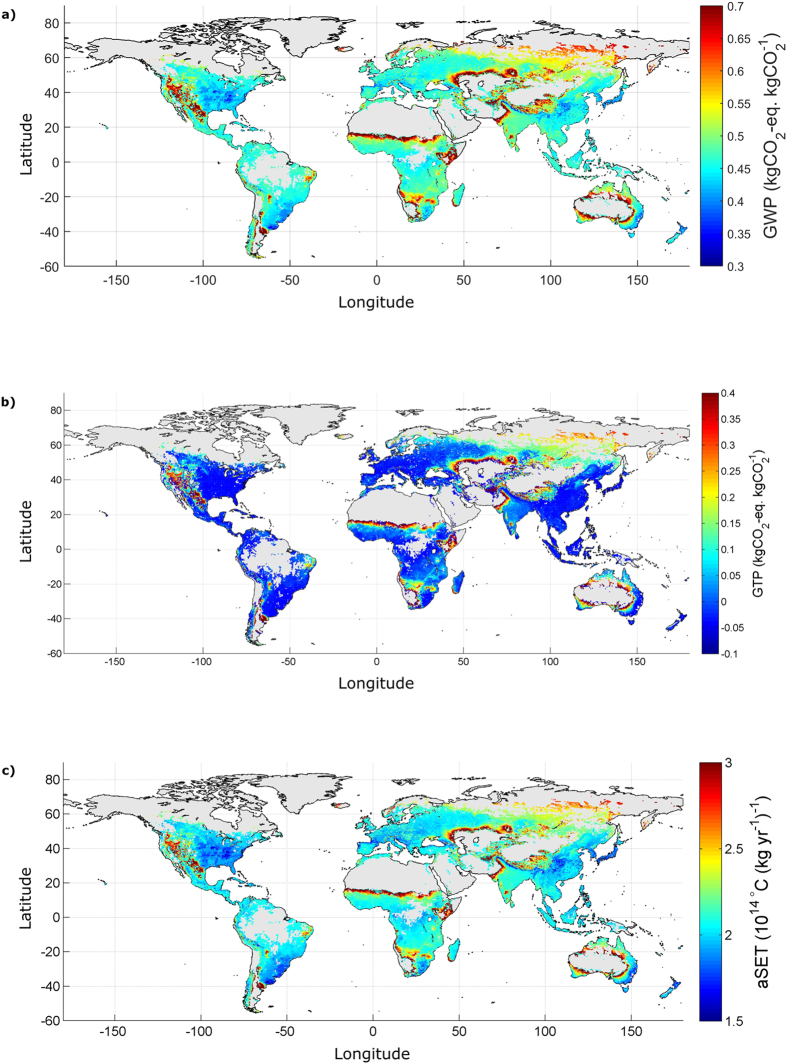
Spatially explicit emission metrics for GWP (TH = 100), GTP (TH = 85), and aSET. GWP (**a**) and GTP (**b**) are in kg CO_2_-eq. per kg of CO_2_ emissions, and aSET (**c**) has units of 10^–14^ °C (kg yr^−1^)^−1^. The results in the figure refer to the case with 50% residue extraction rate. See supplementary Figure S4 for the results under alternative residue extraction rates. Maps are created using MATLAB®.

The spatially explicit emission metrics GWP, GTP, and aSET are presented in Fig. 1. At a global level, we calculate a GWP (Fig. 1a) of 0.49 ± 0.03 kgCO_2_-eq. kgCO_2_^−1^ (mean ± standard deviation), a GTP (Fig. 1b) of 0.05 ± 0.05 kgCO_2_-eq. kgCO_2_^−1^, and an aSET (Fig. 1c) of 2.14·10^−14^ ± 1.11·10^−15^ °C (kg yr^−1^)^−1^ for a case in which 50% of harvest residues are extracted. The 5^th^ and 95^th^ percentile variation spans between 0.43 and 0.62 kgCO_2_-eq. kgCO_2_^−1^ for GWP, −0.03 and 0.24 kgCO_2_-eq. kgCO_2_^−1^ for GTP, and 1.90·10^−14^ and 2.64·10^−14^ °C (kg yr^−1^)^−1^ for aSET. As observed in previous studies^19^,^30^,^37^, the metrics are proportional to the amount of forest residues left in the forest. Under idealized conditions of 100% or 0% residue extraction rates, the global mean values become 0.41 ± 0.02 and 0.65 ± 0.05 kgCO_2_-eq. kgCO_2_^−1^ for GWP, 0.04 ± 0.04 and 0.07 ± 0.07 kgCO_2_-eq. kgCO_2_^−1^ for GTP, and 1.81·10^−14^ ± 8.94·10^−16^ °C (kg yr^−1^)^−1^ and 2.81·10^−14^ ± 1.85·10^−15^ °C (kg yr^−1^)^−1^ for aSET, respectively.

GTPs generally have lower values than GWPs, with negative values resulting from the fast time scale of atmospheric-ocean CO_2_ exchange relative to the growth cycle of biomass. This mechanism is described in previous studies^20^,^21^ and highlighted by the 5^th^ IPCC assessment report^16^. Higher metric values are observed in cold biomes, such as boreal and mountainous areas, and in semi-arid regions, such as in proximity to deserts and savannahs. Emission metrics are comparably lower in the subtropical climate domain, notably south-eastern Asia, southern Brazil, and south-eastern US, in which favourable climate conditions and species selection promote high forest productivities. Earth observation satellite data of global forest cover changes indicate that these regions experience extensive forestry land uses, in which forests are often managed with short rotation times and the presence of long-lived forest plantations is relatively rare^38^.

**Figure 2:**
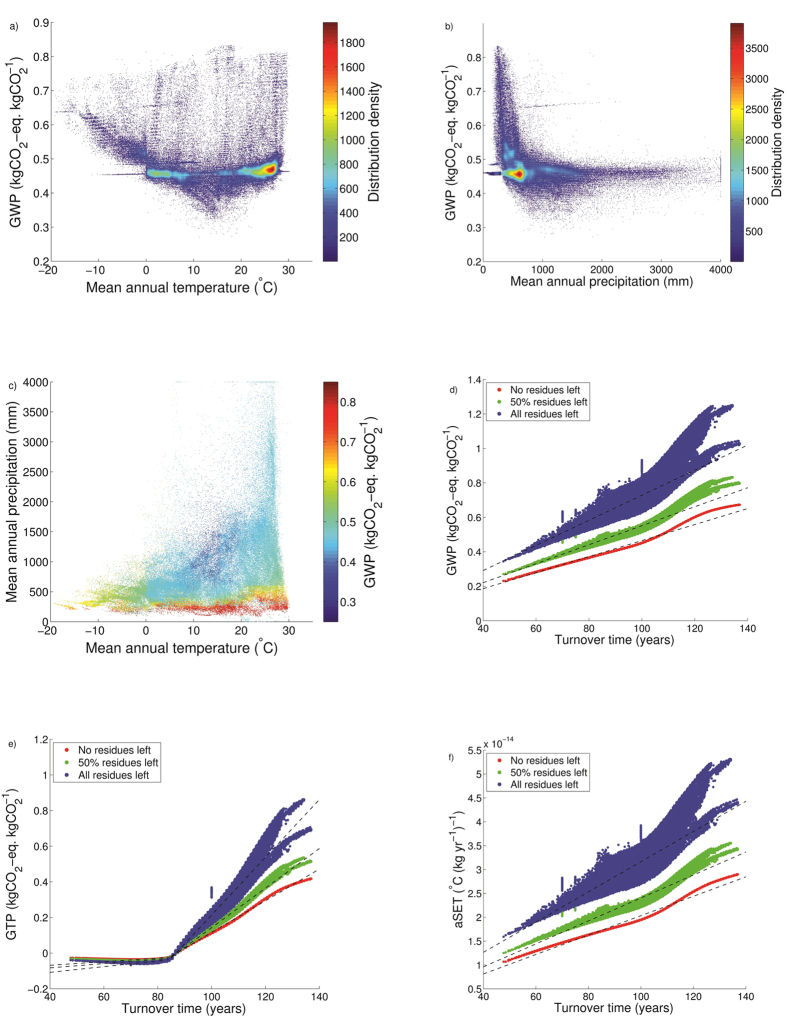
Relationship of emission metrics with climate variables and turnover times. GWP values (50% forest residue extraction) are presented as a function of the mean annual temperature (**a**), mean annual precipitation (**b**) and both (**c**). The colour bar in (**a**,**b**) refers to the distribution density of the GWP scores, defined as the number of points in the proximity of each point within a predefined range (10% of the corresponding maximum value, that is 0.4 °C for temperature, 40 mm for precipitation and 0.008 for GWP). The colour bar in (**c**) indicates the GWP value. The relationship between emission metrics and turnover times τ (that is, the rotation period of the plantation) is presented in (**d**) for GWP (TH = 100), (**e**) for GTP (TH = 85), and (**f**) for aSET, according to different forest residue extraction rates (0%, 50% and 100%). Dashed lines represent the linear fit. The equations and associated R^2^ and RMSE values are as follows: (**d**) 50%: GWP = 0.0055·τ (R^2^ = 0.835; RMSE = 0.030); no residues: GWP = 0.0046·τ (R^2^ = 0.863; RMSE = 0.022); all residues: GWP = 0.0073·τ (R^2^ = 0.738; RMSE = 0.060). (**e**) 50%: GTP = 0.0012·τ-0.131 (R^2^ = 0.744; RMSE = 0.004) for τ ≤ 85 and GTP = 0.011·τ-0.967 (R^2^ = 0.990; RMSE = 0.012) for τ > 85; no residues: GTP = 0.001·τ-0.108 (R^2^ = 0.749; RMSE = 0.003) for τ ≤ 85 and GTP = 0.00089·τ-0.774 (R^2^ = 0.996; RMSE = 0.006) for τ > 85; all residues: GTP = 0.0016·τ-0.172 (R^2^ = 0.749; RMSE = 0.006) for τ ≤ 85 and GTP = 0.0162·τ-1.406 (R^2^ = 0.990; RMSE = 0.032) for τ > 85. (**f**), 50%: aSET = 2.40E-16·τ (R^2^ = 0.781; RMSE = 1.32E-15); no residues: aSET = 2.03E-16·τ (R^2^ = 0.805; RMSE = 1.01E-15); all residues: aSET = 3.16E-16·τ (R^2^ = 0.712; RMSE = 2.46E-15).

In Fig. 2, we investigate the dependence of emission metrics on climate variables and biomass turnover times (here intended as the time required to replenish the biomass resource pool, i.e. the rotation period of the plantation). The temperature and GWP are negatively correlated for average temperatures of less than approximately 15 °C (Fig. 2a) and when precipitation is less than approximately 1000 mm (Fig. 2b). Thereafter, the correlation becomes slightly positive for temperature and displays no clear trend with precipitation. Although the GWP vs. mean annual temperature correlation is highly variable, the majority of the values are around 0.45 ± 0.05 kgCO_2_-eq. kgCO_2_^−1^, with a density maximum at approximately 25 °C. In the GWP vs. precipitation correlation, the highest density of points occurs between 500 and 1000 mm. The lowest GWP values occur around a mean annual temperature between approximately 10 and 20 °C and for annual precipitation greater than approximately 1000 mm (Fig. 2c). This result can be explained by the generally more favourable growing conditions under mild and wet climates. Across all temperature ranges, lower precipitation is usually associated with higher GWP scores, reflecting the fact that moisture is the dominant environmental control of ecosystem productivity^39^,^40^. Temperature control is only relevant at higher precipitation levels, as shown by the more confined data distribution for the precipitation plot (Fig. 2b) compared to the temperature plot (Fig. 2a). The spatial distribution of GTP and aSET follows similar patterns as those for GWP. The geographical variation of emission metrics reflects the distribution of carbon turnover times in global land ecosystems (Fig. 2d–f), which are clearly dependent on temperature and precipitation^40^. The correlation is stronger when all residues are extracted and becomes weaker when the residue extraction rate decreases. This occurs because the post-harvest NEE profiles are more sensitive to the specific R_h_ flux when residues remain in the forest to decompose. In this case, the spatial variability of the metrics increases as the decomposition rate of dead organic biomass is sensitive to the local climate. The differences in the slope of the linear fits thus reflect the additional contribution resulting from the decomposition of forest residues remaining on site after harvest. Whereas GWP and aSET exhibit a single linear correlation, the instantaneous metric GTP is more dependent on the TH and exhibits two different trends. The GTP remains relatively constant to slightly negative for turnover times of up to approximately 85 years, and thereafter, the GTP linearly increases with longer turnover times. The trends in GWP, aSET, and, in part, GTP are similar because each emission metric generally reflects identical relative scores among the different cases. The linear equations in the caption of Fig. 2 can be used to predict the values of the metrics on the basis of the site-specific turnover time and forest residue extraction rate.

**Figure 3:**
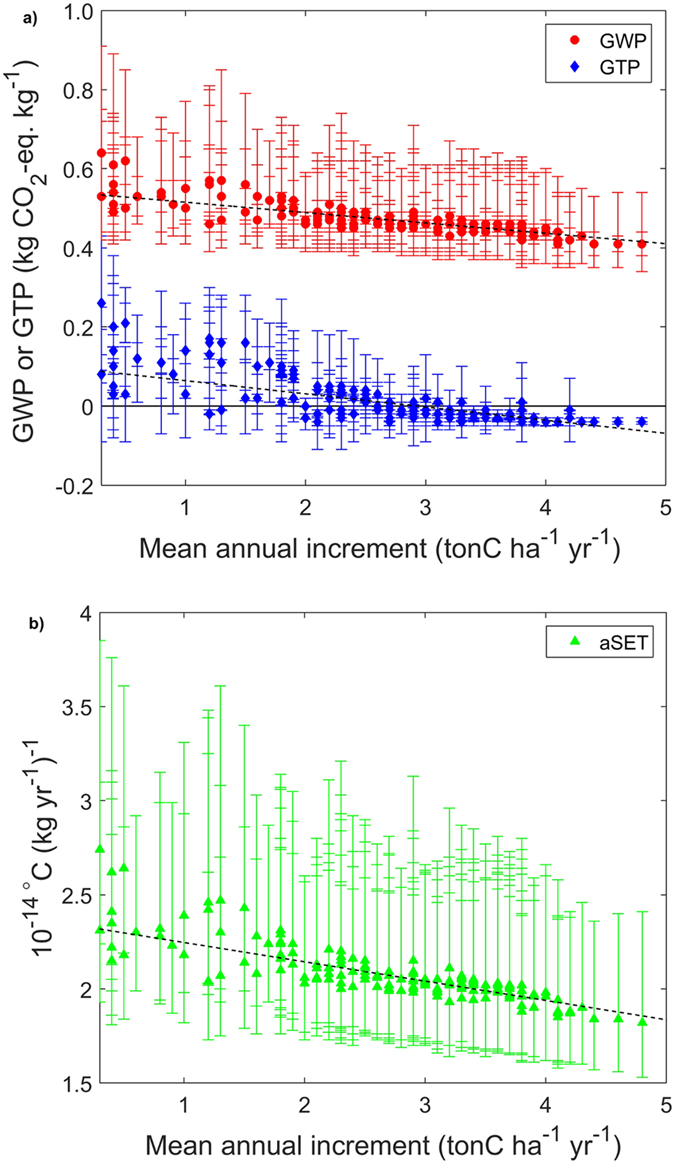
Country average emission metrics as a function of mean annual increment (MAI) of the respective forest areas. (Figure 3a) GWP (TH = 100) and GTP (TH = 85). (Figure 3b) aSET. The mean values represent the ensemble means of the case in which 50% of forest residue are extracted, and the error bars show the respective range given by the mean value for the 100% (higher end) and 0% (lower end) cases of forest residue left in the forest after harvest. The dashed lines represent the linear fit of the ensemble means for the 50% residue extraction case. Equations, R^2^ and RMSE values are as follows: GWP = −0.026·MAI + 0.541 (R^2^ = 0.523, RMSE = 0.029), GTP = −0.033·MAI + 0.098 (R^2^ = 0.398, RMSE = 0.049), aSET = −0.102E-14·MAI + 2.35E-14 (R^2^ = 0.551, RMSE 1.09E-15).

The results aggregated at a country level are plotted against the mean annual increment (MAI) in Fig. 3. The ensemble means refer to a rate of forest residue extraction of 50%, and the error bars refer to the cases of 100% (higher end) or 0% (lower end) residue remaining in the forest after harvest. A detailed statistical analysis with national averages, standard deviations, and 5^th^/95^th^ percentiles is available in Supplementary Table S2. The values of the metrics generally decrease at increasing MAI, which is higher in countries with more favourable growing conditions that can sustain shorter turnover times. GTP displays negative values for countries in which the MAI is larger than approximately 3 tons carbon per hectare per year. These results are in line with the few metric values reported in the scientific literature. For instance, the GWPs computed for a Norwegian forest plantation are estimated to be 0.44, 0.52 and 0.62 kgCO_2_-eq. per kg CO_2_ for residue extraction rates of 0%, 50% and 100%^37^, respectively. These figures are consistent with the mean GWP values aggregated at a national level found in this study (0.44, 0.52 and 0.69 kgCO_2_-eq. per kg CO_2_). Similarly, the GWP of 0.42 kgCO_2_-eq. per kg CO_2_^22^ computed using empirical measurements of post-harvest NEE fluxes in a Canadian pine forest^13^ falls within one standard deviation of the national average (0.45 ± 0.04).

### Applications to forest bioenergy emissions

**Figure 4:**
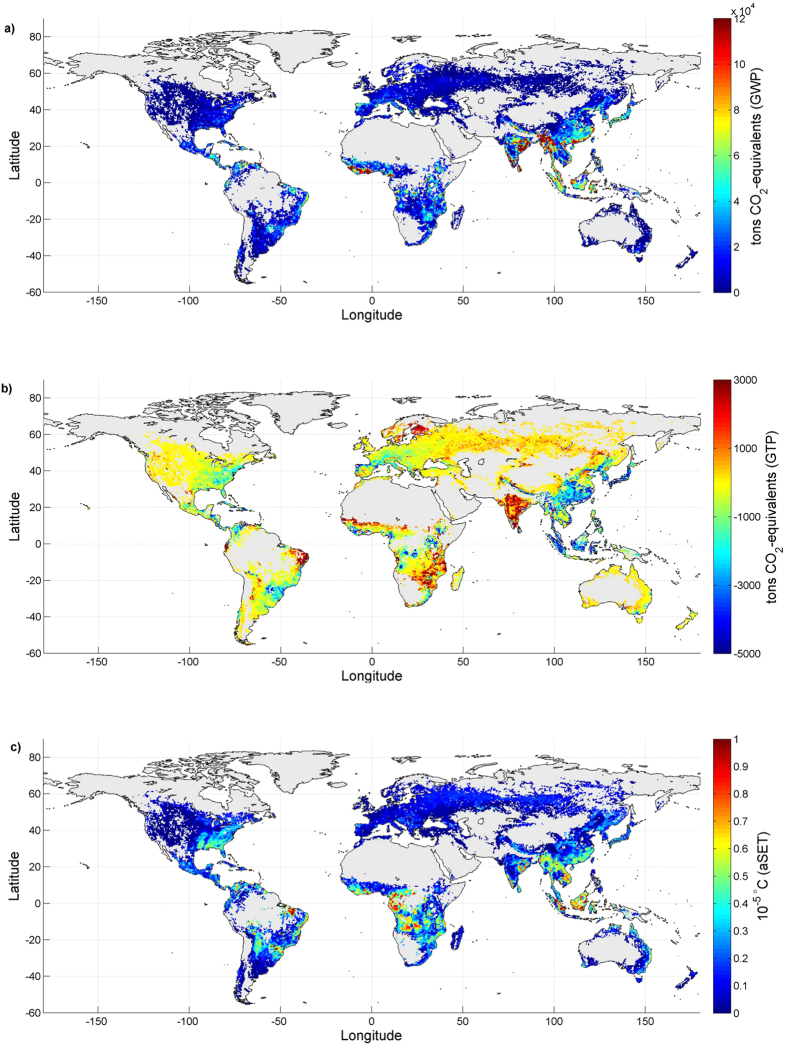
Spatially explicit contributions to the global climate change impacts of the forest bioenergy CO_2_ emissions in the RCP8.5 scenario. GWPs (TH = 100) (**a**) or GTPs (TH = 85) (**b**) are applied to the emissions in 2015. The maximum emission rates in each grid cell from 2015 to 2100 are used for the climate change impact assessment with aSET (**c**). Units are ton CO_2_-equivalents in (**a**,**b**) and 10–5 °C in (**c**). These maps refer to the case in which 50% of forest residues are extracted. See Supplementary Figure S6 for the results under different extraction rates. Maps are created using MATLAB®.

We apply these spatially explicit emission metrics to the global CO_2_ emissions from forest bioenergy that are compatible with the RCP 8.5^35^ scenario, for which annual gridded CO_2_ emissions up to 2100 are available from the land-use harmonization project^36^. Figure 4 shows the results when GWP (Fig. 4a) and GTP (Fig. 4b) are used to assess the climate change impacts of bioenergy CO_2_ emissions from global forests in 2015. The metric aSET (Fig. 4c) is applied to the maximum emission rates occurring in each grid cell from 2015 to 2100 (see Methods). Maps with CO_2_ emission flows (Figure S5) and the climate change impacts under varying extraction rates of forest residues (Figure S6) are available in the supplementary information. At the global level, CO_2_ emissions from forest bioenergy in 2015 correspond to 1.36 (−0.22/+0.44) Gt CO_2_-equivalents using GWPs. By contrast, we calculate a global cooling contribution in 2100 of −0.037 (−0.0025/+0.002) Gt CO_2_-equivalents using GTPs. With the aSET metric, we quantify a contribution to the temperature peak from the entire scenario of 0.17 (−0.3/+0.6) °C. In comparison, the gross CO_2_ emission flows in 2015 amount to 2.99 Gt, and the contribution to the temperature peak would be 0.26 ± 0.07 °C if the emissions from 2015 to 2100 stemmed from fossil fuels. The major forest bioenergy producing regions in 2015 are Southeast Asia, the Indian Peninsula, and Southeast Africa. When GWPs are used (Fig. 4a), these regions display positive contributions in terms of CO_2_-equivalents. This situation changes when GTPs are used as the emission metric (Fig. 4b). In Southeast Asia, many grids have GTP values of less than zero (see Fig. 1b), thus yielding negative contributions to the global temperature in 2100 for CO_2_ emissions from bioenergy sourced from forest biomass located in this region. This cooling effect, together with the effects from other locations across the globe, more than offsets the warming contribution from the Indian Peninsula and Southeast Africa, in which relatively high emission flows are combined with GTP factors that are typically higher than zero. The geographical distribution of emissions from bioenergy changes over the 21^st^ century. A substantial growth in bioenergy production is predicted in regions such as the south-eastern US, South America and sub-Saharan Africa, and this growth is reflected in the higher contribution to the temperature peak from these areas (Fig. 4c). The interpretation of the results and the relative ranking of the contributing regions are largely dependent on the climate change aspect represented by the selected emission metric. For instance, at the aggregated continental level (Table 1, see Supplementary Table S3 for country data), whether GWP or GTP is used, Asia transitions from the largest contributor to global warming to the main contributor to global cooling. Similarly, Europe transitions from a global warming of 118 (−17/+33) Mt CO_2_-equivalents (with GWP) to nearly climate neutral (with GTP).

## Discussion

The large differences in the results after applying GWPs or GTPs stem from the different characteristics of the two metrics. We recall that GWP is a time-integrated measure that considers the total forcing added to the climate system until the TH. The corresponding results in Fig. 4a are not indicative of an equivalence with temperature. On the other hand, the GTP specifically establishes a comparison in terms of instantaneous temperature change at the desired TH^27^. By adopting a TH of 85 years, our results reveal an average negative (cooling) contribution of global forest bioenergy emissions in 2015 relative to the global average surface temperature in 2100. In most of the cases, the TH is set once the warming perturbation has nearly ceased. The results are sensitive to the TH or to the year in which emissions occur, notably when based on instantaneous metrics such as the GTP. For shorter THs or emissions occurring in proximity to the target, the GTP values gradually increase because the TH would fall before the warming contribution has dissipated. The aSET metric is still based on temperature, but it considers a different dimension of climate change than GTP. The aSET metric is used to infer the temperature peak from bioenergy CO_2_ emission scenarios, which is proportional to the maximum rate at which emissions occur and is insensitive to the total amount of emissions. This metric does not explicitly capture the timing of the different temperature peaks and post-peak dynamics, which significantly differ between short- and long-lived climate forcers. For CO_2_ emissions from bioenergy systems, the effect gradually dissipates over time, and the temperature decreases if emission rates decrease^20^. For long-lived GHGs such as CO_2_ from fossil fuels, the effect persists over time, and the temperature continues to increase for each additional unit of emission and does not decline even after the emissions cease^41^,^42^. The temperature will eventually stabilize at a near-peak level only when the emission rates decrease to zero^28^,^43^.

This study is the first to offer global spatially explicit emission metrics for forest bioenergy. The metrics are formulated following the same IPCC approach that is commonly applied for other GHGs and thus allow to aggregate climate impacts from bioenergy CO_2_ emissions to those from other forcing agents into common units. The values of the metrics reflect regional differences in climate and forest management. The maps have geographical patterns that relate to local climate variables and forest characteristics. Metrics are generally lower in regions with favourable growing conditions and when forest residues are extracted rather than left to oxidize on the plantation ground. On the other hand, we find relatively high emission metrics with low precipitation, long rotation times and low residue extraction rates. The availability of this dataset is a timely and easy-to-use option that offers the possibility to integrate the climate change contributions of CO_2_ emissions from forest bioenergy with the variety of applications in which emission metrics are used. Applications can span from local projects to global emission scenarios, in which the role of forest bioenergy can be assessed under different objectives using the related climate change impact indicators.

Emission metrics have been successfully applied to illustrate the basic physics of the response of the climate system to emissions and are widely used in the scientific community and legislative frameworks. However, there are a number of uncertainties and limitations associated with metrics that are primarily connected to the definition of background climate conditions and the treatment of feedbacks^29^. The IPCC standard protocol for emission metrics defines a constant background climate and excludes possible feedbacks of climate change into the terrestrial carbon cycle. Future climate change and increasing atmospheric CO_2_ are expected to cause substantial changes in vegetation structure and function over large fractions of the global land surface. Understanding the net effect of climate feedbacks on terrestrial vegetation is complex because of the variety of factors involved. Changes in NPP, R_h_ and NEE occur primarily through enhanced growth because of CO_2_ fertilization effects, nitrogen availability, faster decay rates of residues, and higher soil respiration^44^,^45^,^46^. Recent modelling studies investigating the overall global effect of projected future climate change on terrestrial vegetation indicate large heterogeneity in terms of the magnitude and sign of the net change in NEE, and generally conclude that the response of the terrestrial carbon cycle to climate change is one of the largest sources of uncertainty affecting future climate change projections^47^,^48^,^49^. The possible effects of a changing climate on emission metrics for forest bioenergy can be qualitatively estimated via the dependencies of metrics on temperature and precipitation (Fig. 2). Metrics will gradually decrease in areas experiencing generally more favourable growing conditions, whereas metrics should increase in regions in which climate change is expected to exacerbate drought periods. Future growth in CO_2_ atmospheric concentrations will also increase the fraction of CO_2_ remaining airborne over time because of saturation in the oceans and land carbon stocks^29^ and simultaneously decrease the marginal radiative efficiency of CO_2_^16^. However, emission metrics are relatively insensitive to these effects because the saturation of carbon sinks is nearly offset by the saturation of CO_2_ radiative forcing at increasing atmospheric CO_2_ concentrations^29^,^50^,^51^.

Following our approach, alternative emission metrics can be computed. For instance, GWPs and GTPs can be based on a different TH, or other climate impact indicators can be considered, such as sea level rise^52^ or global precipitation changes^53^. A future multi-model inter-comparison project is desirable to further assess emission metric dependencies on model parameterizations, climate conditions, and vegetation structure. There is an increasing number of different models available for this purpose. Other stand-alone global land and vegetation models can track secondary forest regrowth and capture the influence of wood harvesting on carbon fluxes^36^,^54^,^55^. Integrated assessment models also account for land-use emissions, including those from bioenergy^4^,^56^,^57^. The representation of land-use dynamics is also continuously improving in global climate models^56^,^57^,^58^,^59^, including earth system models, in which the effects from forest management are usually difficult to assess because of the limited representations of forest successional stages and management practices^59^. Further research developments can also produce extensive empirical measurements for the post-harvest NEE dynamics. A global tree species map with the associated information on tree components at different successional stages would enable an improvement of the representation of NEE fluxes and a calibration of global vegetation models. Although some valuable steps in this direction have been made^39^, the creation of such a map would require large and standard measurements of biomass compartments because many important tree species across different climate zones and age classes must be covered. A possible extension of this work is the formulation of emission metrics for forest harvest materials that are used as wooden products in our society^60^. Such metrics can be combined with the country average lifetimes of the products^61^ to obtain national aggregated estimates. Aggregated country values are suitable for incorporation into global multi-regional input output tools used for carbon footprint calculations, and maps of emission metrics can be complementary to integrated assessment studies to infer the climate change implications of different forest bioenergy options. Emission metrics for forest bioenergy can also be included into impact assessment methods as characterization factors for routine applications in the field of LCA and environmental impact analysis in general. Using the simplified linear regressions from Fig. 2, the users are left to specify the turnover time of the plantation and the fraction of forest residues extracted. Changes in forest cover affect the delivery of important climate regulating services other than CO_2_, such as surface reflectivity (albedo), water fluxes, and surface roughness^62^,^63^. These so-called biogeophysical effects are not of secondary importance because, in some cases, they can more than offset the climate forcing associated with the net changes in CO_2_ fluxes^64^,^65^. In principle, spatially explicit maps of the responses to these non-CO_2_ climate forcing mechanisms following a forest disturbance can be produced^66^, and future research can explore strategies to integrate them with carbon-based indicators.

This work reflects the complex interactions between forest ecosystem responses, climate change, and the role of the selected metric used to inform policy. In-depth multidisciplinary research between foresters, climate scientists and emission scenario developers will further consolidate a consistent understanding of the implications associated with forest resource use in the context of climate change mitigation.

## Methods

### Global Forest Model (G4M)

G4M is a geographically explicit vegetation model that simulates global forest characteristics and is frequently used to model forest carbon dynamics associated with human-induced disturbances^24^,^31^,^32^,^67^. The global forest characteristics from G4M used as the basis for this analysis are presented in supplementary Figure S1. In G4M forest growth is determined by a potential Net Primary Productivity (NPP) estimate which is based on temperature, precipitation and soil characteristics. A stocking density in each cell is initialized in an iterative procedure that combines the observed stocking biomass from the Global Forest Resource Assessment statistics^5^, and net annual increment per grid cell^31^,^67^. The stocking degree and the biomass turnover times are influenced by human activity, described by layers of human population pressure, and are based on forest management activities that maximize increments^32^. We model secondary forest plantations by masking the wilderness and non-productive areas^15^, and we filter out the grids in which the fraction of primary forest exceeds 90%. Post-harvest NPP dynamics are modelled in each grid cell by considering the system as net carbon neutral along the site-specific turnover time, although carbon gains or losses are possible on a case-specific basis. G4M estimates the fraction of woody residues at harvest through a simplified look-up table, which is improved by considering the average woody and non-woody components as a function of the diameter at breast height (see Supplementary Table S1). Bark, foliage and fine roots are assumed to be non-woody components. Supplementary Figure S2 presents the spatial distribution of the amount of woody and non-woody forest residues at harvest per grid cell. We use the WorldClim database for global climate parameters (maximum, minimum, and mean temperature and precipitation) that are representative of current conditions (interpolation of observed data from 1950)^34^.

### Heterotrophic respiration (R_h_)

The R_h_ response to the harvest event is obtained from reproducing YASSO07^33^ via a statistical model reduction that can estimate the decomposition rate of residues with climate-explicit variables such as the mean annual temperature, average annual precipitation, and mean amplitude of average monthly minimum and maximum temperature. We perform the YASSO07 runs assuming the soil carbon stock at a steady state at the beginning of the simulation, and carbon inputs to the soil are added as a unit pulse at year zero to simulate the input of forest residues to the dead organic matter component. This process allows the resulting R_h_ response to be scaled to the grid-specific amount of forest residues remaining on site after harvest. Post-harvest net CO_2_ exchanges are estimated for each grid cell after combining the NPP and R_h_ profiles. These NEEs represent the ecosystem carbon response to the harvest event and are used as the basis for the computation of emission metrics for forest bioenergy^18^,^22^,^23^. The main results presented in this paper refer to a forest residue extraction rate of 50%, and idealized cases of extraction rates of 0% or 100% are explored as extreme options to mark the higher and lower uncertainty bounds of the expected range of emission metrics. Whereas the influence of forest residues on heterotrophic respiration is considered, possible feedbacks on NPP or soil fertility are not accounted for in the high residue extraction cases.

### Emission metrics

Emission metrics are computed following the standard protocol^29^ used by the 5^th^ IPCC Assessment Report^16^. The change in CO_2_ atmospheric concentration from bioenergy CO_2_ emissions and the associated site-specific NEE profiles are computed for each grid cell after the integration with the global carbon cycle through a mathematical convolution^22^:


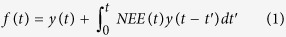


where *f(t)* is the grid-specific impulse response function of the perturbation and *y(t)* is the impulse response function to a CO_2_ emission, simulated using a multi-model mean^29^. This value is then translated into radiative forcing (RF) using the radiative forcing expression for CO_2_^68^:


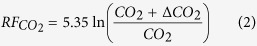


where CO_2_ is the background atmospheric CO_2_ concentration (equal to the average concentration in 2010 of 389 ppmv^29^) and ΔCO_2_ is the change from the reference state induced by the pulse emission. The increase in RF following a unit (kg) increase in the atmospheric abundance of CO_2_, called the radiative efficiency, 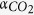, is given by the following:


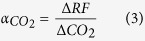


The instantaneous RF is then computed as the product of equation 1 and equation 3. The time-integrated RF is the absolute global warming potential (AGWP, see Supplementary Figure S3a) used to calculate the GWP metric^69^.

The global surface temperature responds to changes in radiative forcing on a spectrum of timescales. We use a temperature response function that is the sum of two exponentials:


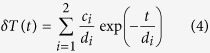


The sum of the coefficients *c*_*i*_ is the climate sensitivity, and *d*_*i*_ represents two different timescales. We use the factors *c*_*1*_ = 0.631 K/(Wm^2^), *c*_*2*_ = 0.429 K/(Wm^2^), *d*_*1*_ = 8.4 yr, and *d*_*2*_ = 409.5 yr from a previous publication^70^, which correspond to an equilibrium climate sensitivity of 1.06 °C/(Wm^2^) (or 3.9 °C for CO_2_ doubling). The temperature response to a radiative forcing pulse, called the Absolute Global Temperature Change Potential (AGTP, see Supplementary Figure S3b), is then estimated as follows:


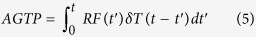


The GTP metric (computed as described in ref. 69) is the ratio between the AGTP of the temperature response to the bioenergy system in the specific grid and the AGTP for CO_2_ at a given TH. Supplementary Figure S3 shows the spatially averaged temporal dynamics of the AGWP and AGTPs across all grids.

The metric aSET is specific for short-lived climate forcers, for which the contribution to the temperature peak is proportional to the maximum rate at which emission occurs. This metric is developed to assess climate change contributions under a two-basket approach in which GHGs are differentiated as long- and short-lived and is computed following the method described in ref. 28.

### Fuelwood case study

Spatially explicit emission metrics are applied to the wood harvest emissions for global forest bioenergy. We use the gridded wood harvest data compatible with the RCP8.5 scenario^35^ from the land-use harmonization project that provide the annual biofuel harvest per grid cell in tons of emitted carbon up to 2100^36^. No annual gridded information on forest harvest for bioenergy is available for the other RCPs.

The characterized results are computed for all the grids *n* as follows:


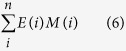


where *E(i)* is the CO_2_ emission flow from forest bioenergy in grid cell *i* and *M(i)* is the corresponding emission metric. For GWP and GTP, *E(i)* are the emissions in 2015 (Supplementary Figure S5a), and the results are in kg CO_2_-equivalents. In the case of aSET, *E(i)* is the maximum emission rate from 2015 to 2100 occurring in each grid cell (Supplementary Figure S5b), and the results refer to the temperature peak contribution (in °C). The wood harvest scenario envisages emissions from grid cells that are masked in our study because they were identified as wilderness areas or forest regions dominated by primary forests^15^. These emissions are therefore excluded from our analysis. To quantify the contribution to the temperature peak if these emissions were sourced from fossil fuels, we sum the emissions from each grid cell until 2100 (Supplementary Figure S5c) and then use the multi-model mean transient response to cumulative emissions of 1.75 ± 0.4 °C per TtC emitted to estimate the effect on the temperature peak^43^.

## Additional Information

**How to cite this article**: Cherubini, F. *et al.* Global spatially explicit CO_2_ emission metrics for forest bioenergy. *Sci. Rep.* **6**, 20186; doi: 10.1038/srep20186 (2016).

## Supplementary Material

Supplementary Information

## Figures and Tables

**Table 1:** Climate change impacts aggregated at a continental level after applying the emission metrics GWP (TH = 100), GTP (TH = 85), and aSET to the global CO_2_ emissions from forest bioenergy in RCP8.5.

Continent	CO_2_ emissions from forest bioenergy			GWP	Rang	GTP	Range	aSET	Range
	2015 (Mt)	Max rate (Gt yr^−1^)	Cumulative (Gt)	Mt CO_2_-eq.		Mt CO_2_-eq.		10^−^ °C	
Asia	1502	3.20	200	679	+230/−114	−24.6	−4.1/+2.3	58.7	+21/−10.5
Europe	261	0.74	48.6	118	+33/−17	−1.76	+0.2/−0.18	13.5	+4.1/−2.1
Africa	663	2.42	126	310	+106/−52	−1.23	+2.7/−1.1	44.9	+16.4/−8.1
North America	221	1.14	74.4	97	+29/−15	−5.46	−1.2/+0.6	19.9	+6.4/−3.3
South America	311	1.69	91.0	141	+42/−22	−3.50	−0.1/+0.1	30.6	+10.3/−5.4
Australia	22.1	0.27	11.2	9.7	+2.8/−1.5	−0.10	+0.08/−0.02	4.94	+1.6/−0.8
Oceania	5.77	0.03	1.73	2.51	+0.8/−0.4	−0.20	−0.06/0.03	0.54	+0.2/−0.1
Total	2986	9.49	553	1357	+444/−222	−36.8	−2.5/2.0	173	+60/−30.4

The table also shows the CO_2_ emissions from bioenergy in 2015, the maximum emission rate and the total cumulative emissions in each grid cell until 2100. The columns GWP, GTP and aSET show the results obtained by applying the corresponding metric (50% residue extraction rate). The range refers to the variation associated with the cases in which 0% or 100% of forest residues are extracted.
