# Supplementary material for: Global spatially explicit CO2 emission metrics for forest bioenergy
Source: Sci Rep. 2016 Feb 2;6:20186. doi: 10.1038/srep20186 (PMC4735754; doi:10.1038/srep20186)
Supplement: Supplementary Information [file srep20186-s1.doc]

Supplementary Information

**Global spatially explicit CO_2_ emission metrics for forest bioenergy**

Francesco Cherubini^1^, Mark Huijbregts^2,3^, Georg Kindermann^4^, Rosalie Van Zelm^2^, Marijn Van Der Velde^5^, Konstantin Stadler^1^, Anders Hammer Strømman^1^

^1^Industrial Ecology Programme, Department of Energy and Process Engineering, Norwegian University of Science and Technology (NTNU), Trondheim, Norway

^2^Department of Environmental Science, Radboud University, Nijmegen, The Netherlands

^3^ Dutch Environmental Assessment Agency, Bilthoven, The Netherlands

^4^Ecosystems Services and Management Program (ESM), International Institute for Applied Systems Analysis (IIASA), Laxenburg, Austria

^5^European Commission, Joint Research Centre (JRC), Ispra, Italy

| 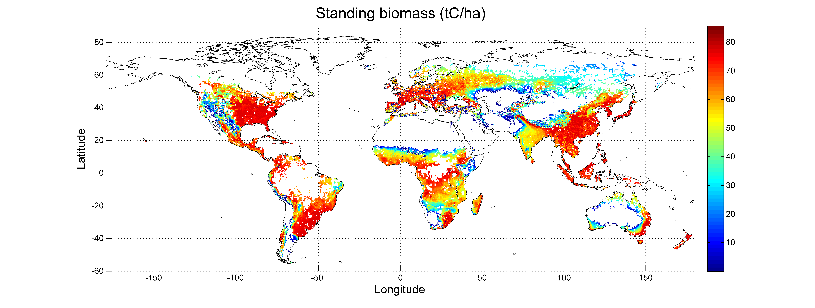  a) | 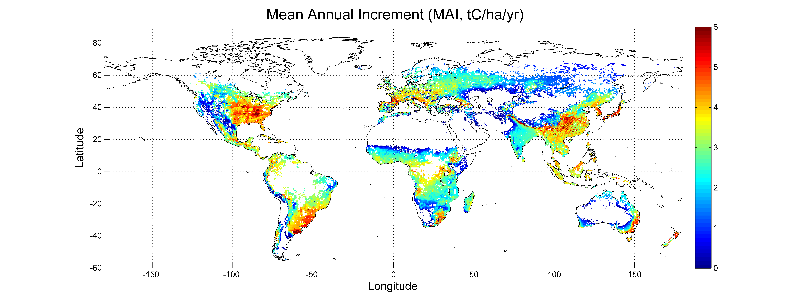  b) |
| --- | --- |
| 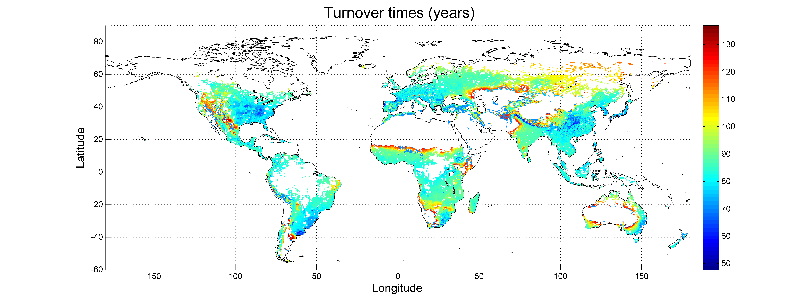  c) | 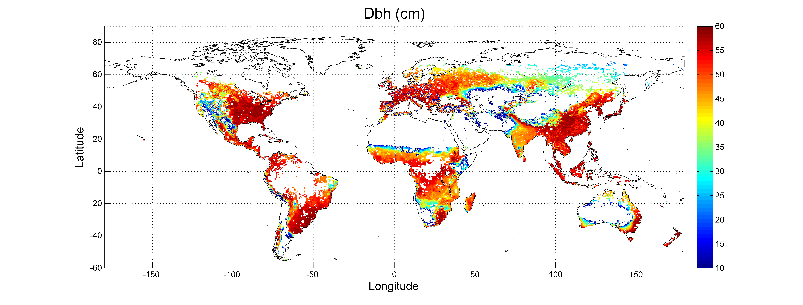  d) |
| 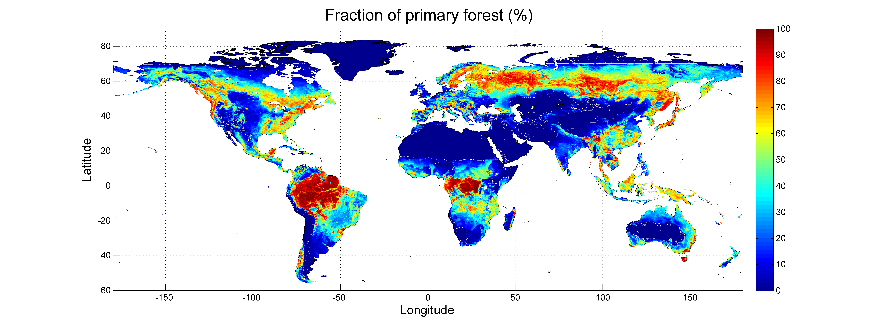  e) | |

Figure S1 Global forest characteristics from the G4M model. The figure shows at a grid cell resolution the average standing biomass (a), the mean annual increment (MAI) (b), the turnover times (c), the diameter at breast height (dbh) at harvest (d), and the fraction of primary forests (e). G4M produces biomass caracteristics for all the globe; in this study, we mask wilderness and desert areas (as identified in ref.^1^) and the grids in which the fraction of primary forest is higher than 90%. Maps are generated using the software Matlab.

Table S1: Look-up table for the average relative fraction of forest residues at harvest as a function of dimater at brest height (dbh)^2^.

| **Dbh (cm)** | **All res** | **Woody components** | **Non-woody components** |
| --- | --- | --- | --- |
| 10 | 0.600 | 0.450 | 0.150 |
| 20 | 0.533 | 0.388 | 0.146 |
| 30 | 0.504 | 0.354 | 0.150 |
| 40 | 0.475 | 0.325 | 0.150 |
| 50 | 0.463 | 0.308 | 0.154 |
| 60 | 0.442 | 0.288 | 0.154 |

| 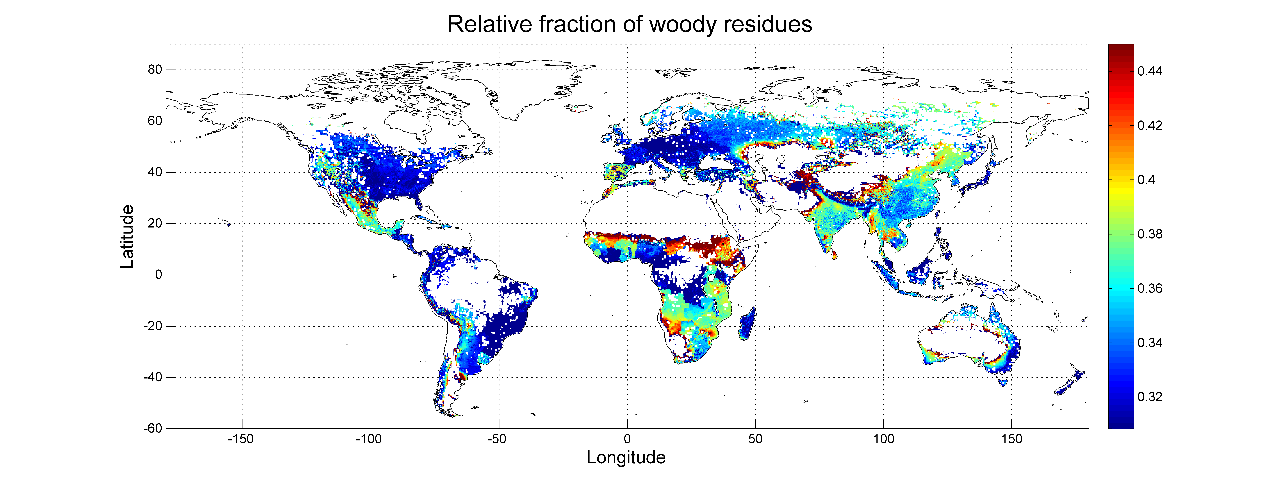  a) |
| --- |
| 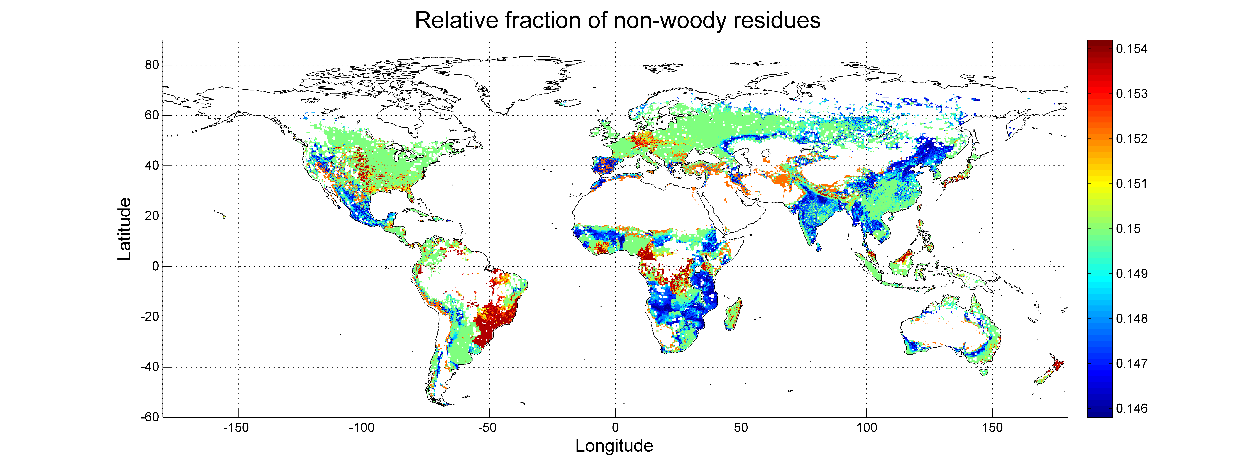  b) |

Figure S2 Spatially explicit values for the fraction of woody (a) and non-woody (b) residues at harvest. Maps are generated using the software Matlab.

| 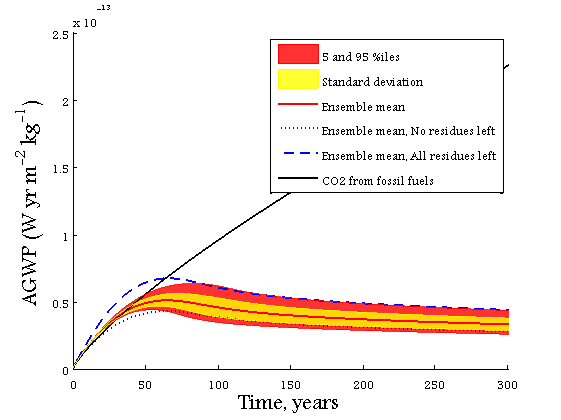  a) |
| --- |
| 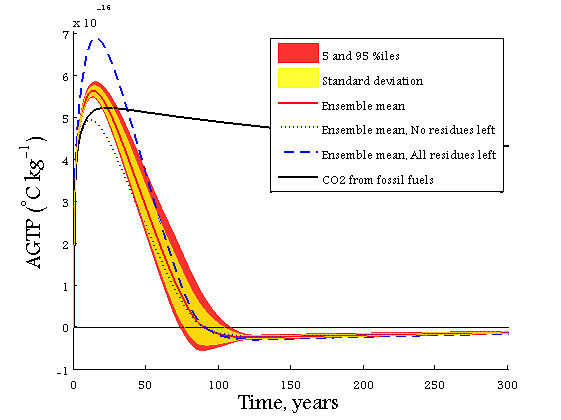  b) |

Figure S3 Absolute Global Warming Potential (AGWP) (a) and Absolute Global Temperature change Potential (b) (AGTP) for the global gridded data. Figure shows the ensemble mean, standard deviation, and 5^th^-95^th^ percentile of the case with 50% forest residue extraction. For the cases with 0 or 100% residues left in the forest only the ensemble means are shown. The curves associated with a pulse emission of CO_2_ from fossil fuels are shown as a benchmark. Following a pulse emission, AGWP is the time-integrated radiative forcing, and AGTP is the global mean surface temperature change^3^. GWP and GTP metrics are given by the ratio between the values of AGWP and AGTP, respectively, at the selected TH of the bioenergy case relative to the fossil case.

| 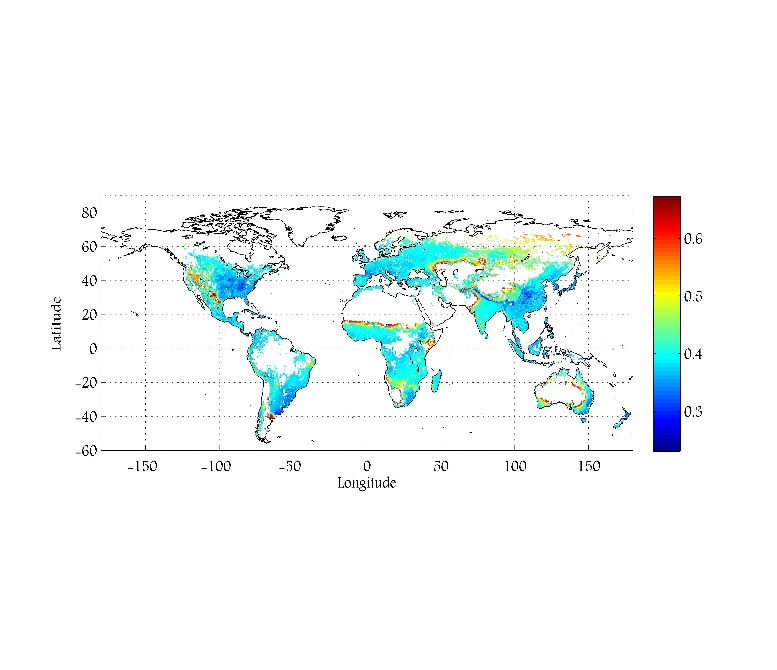 | 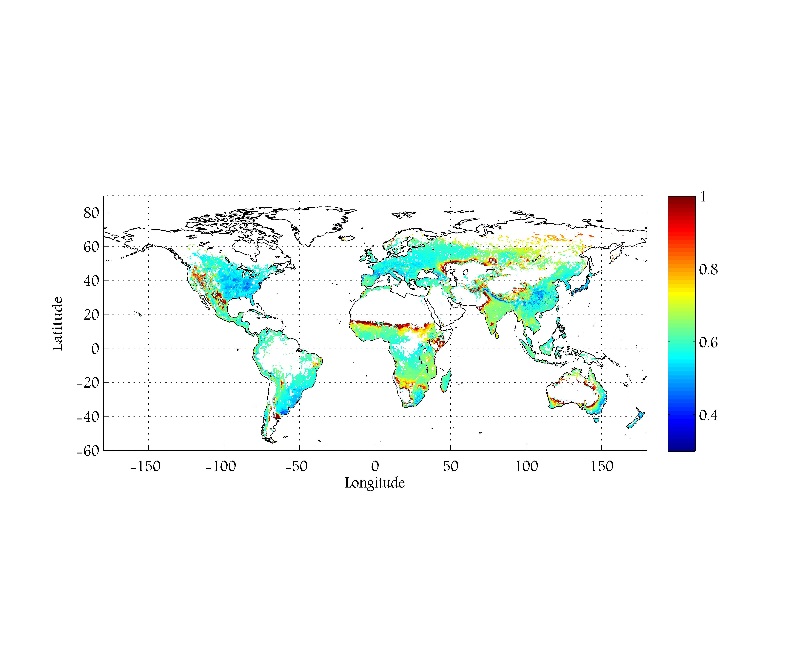  b) |
| --- | --- |
| 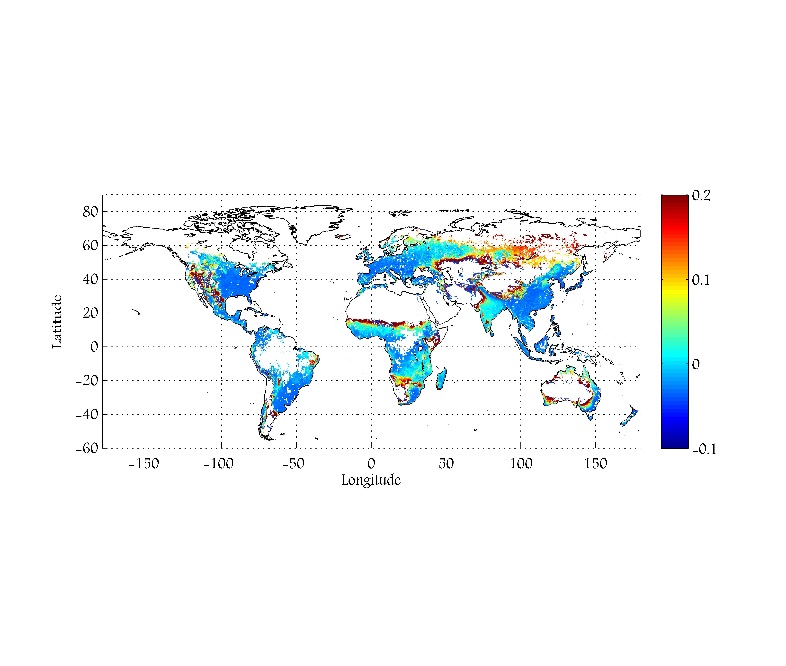 | 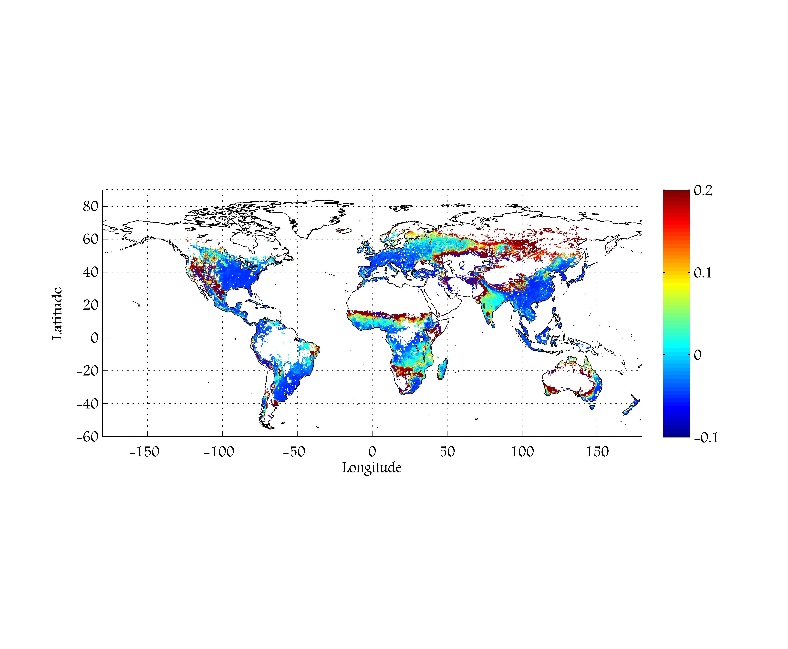  d) |
| 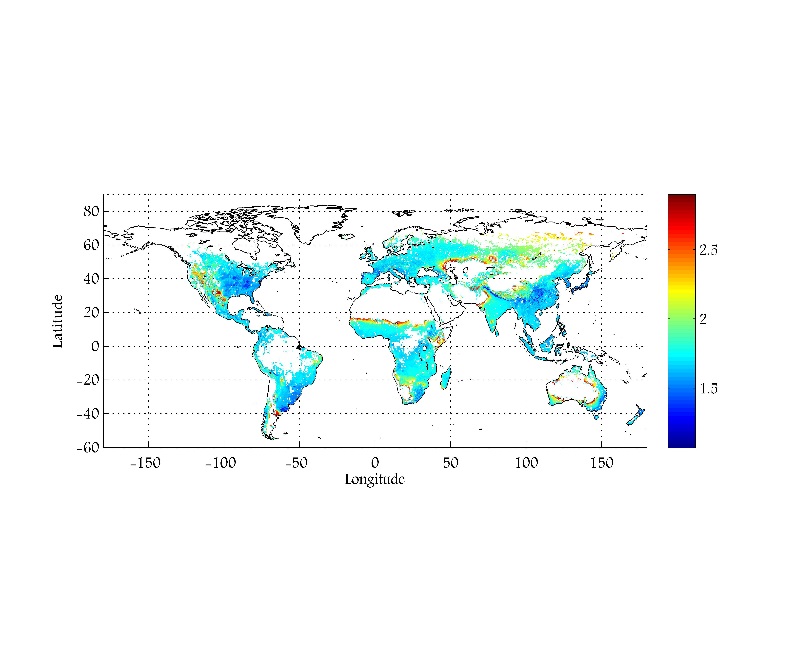  e)  a) | 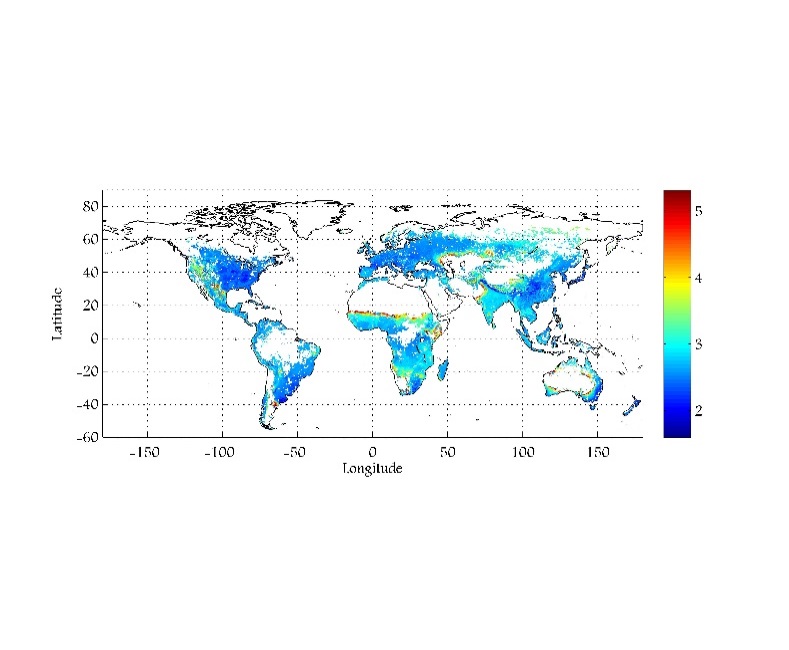  f) |

c)

Figure S4 Spatially explicit emission metrics with 100% (a, c, e) and 0% (b, d, f) residue extraction rates for the emission metrics GWP (a, b), GTP (c, d), and aSET (e, f). GWP and GTP are in kg CO_2_-eq. per kgCO_2_, aSET is in 10^-14^ °C (kg yr^-1^)^-1^. Maps are generated using the software Matlab.

Table S2: Emission metrics aggregated at a national level with the associated statistical analysis. For each country, we report the mean value, standard deviation (σ), 5^th^ and 95^th^ percentile for the 50% forest residue extraction case, and the ensemble means for the cases with no residues (No res) or all residues (All res) left on the field following harvest.

|  |  |  | GWP 100 | | | | | | GTP 85 | | | | | | aSET (10^-14^ °C yr kg^-1^) | | | | | |
| --- | --- | --- | --- | --- | --- | --- | --- | --- | --- | --- | --- | --- | --- | --- | --- | --- | --- | --- | --- | --- |
| COUNTRY | N. of grids | MAI | Mean | σ | 5^th^ %ile | 95^th^ %ile | No res | All res | Mean | σ | 5^th^ %ile | 95^th^ %ile | No  Res | All  Res | Mean | σ | 5^th^ %ile | 95^th^ %ile | No Res | All Res |
| Afghanistan | 854 | 0.4 | 0.54 | 0.11 | 0.37 | 0.78 | 0.46 | 0.72 | 0.10 | 0.18 | -0.05 | 0.45 | 0.080 | 0.15 | 2.35 | 0.41 | 1.69 | 3.28 | 1.99 | 3.10 |
| Albania | 42 | 3.9 | 0.43 | 0.02 | 0.36 | 0.46 | 0.36 | 0.56 | -0.04 | 0.01 | -0.05 | -0.02 | -0.033 | -0.05 | 1.90 | 0.10 | 1.63 | 2.07 | 1.61 | 2.47 |
| Algeria | 342 | 1.3 | 0.47 | 0.03 | 0.42 | 0.78 | 0.40 | 0.61 | -0.01 | 0.06 | -0.05 | 0.48 | -0.009 | -0.01 | 2.07 | 0.11 | 1.88 | 3.31 | 1.75 | 2.70 |
| Andorra | 1 | 0.0 | 0.46 | 0.00 | 0.46 | 0.46 | 0.39 | 0.59 | -0.05 | 0.00 | -0.05 | -0.05 | -0.041 | -0.06 | 2.06 | 0.00 | 2.06 | 2.06 | 1.74 | 2.65 |
| Angola | 1521 | 2.9 | 0.48 | 0.05 | 0.39 | 0.78 | 0.39 | 0.65 | 0.01 | 0.07 | -0.05 | 0.45 | 0.002 | 0.01 | 2.09 | 0.18 | 1.75 | 3.29 | 1.74 | 2.84 |
| Argentina | 4020 | 2.1 | 0.49 | 0.10 | 0.33 | 0.83 | 0.41 | 0.64 | 0.04 | 0.15 | -0.05 | 0.53 | 0.031 | 0.07 | 2.13 | 0.41 | 1.49 | 3.53 | 1.79 | 2.80 |
| Armenia | 46 | 3.1 | 0.45 | 0.05 | 0.37 | 0.64 | 0.39 | 0.59 | 0.01 | 0.07 | -0.05 | 0.27 | 0.005 | 0.02 | 2.00 | 0.20 | 1.67 | 2.69 | 1.72 | 2.58 |
| Australia | 7151 | 1.3 | 0.53 | 0.11 | 0.31 | 0.83 | 0.44 | 0.72 | 0.11 | 0.16 | -0.05 | 0.54 | 0.084 | 0.17 | 2.30 | 0.45 | 1.44 | 3.56 | 1.93 | 3.08 |
| Austria | 151 | 2.9 | 0.46 | 0.07 | 0.40 | 0.75 | 0.40 | 0.59 | 0.00 | 0.10 | -0.05 | 0.44 | 0.001 | 0.01 | 2.04 | 0.27 | 1.79 | 3.18 | 1.75 | 2.59 |
| Azerbaijan | 105 | 2.6 | 0.47 | 0.06 | 0.36 | 0.66 | 0.40 | 0.61 | 0.03 | 0.09 | -0.05 | 0.29 | 0.023 | 0.05 | 2.06 | 0.23 | 1.63 | 2.77 | 1.76 | 2.66 |
| Bahamas | 3 | 3.7 | 0.44 | 0.00 | 0.44 | 0.45 | 0.37 | 0.58 | -0.03 | 0.00 | -0.03 | -0.03 | -0.029 | -0.04 | 1.97 | 0.00 | 1.96 | 1.97 | 1.66 | 2.55 |
| Bangladesh | 185 | 3.3 | 0.47 | 0.01 | 0.45 | 0.50 | 0.38 | 0.65 | -0.03 | 0.01 | -0.05 | 0.02 | -0.023 | -0.04 | 2.07 | 0.03 | 1.99 | 2.19 | 1.69 | 2.87 |
| Belarus | 431 | 3.1 | 0.45 | 0.01 | 0.43 | 0.47 | 0.39 | 0.56 | -0.02 | 0.01 | -0.03 | 0.04 | -0.016 | -0.02 | 1.98 | 0.03 | 1.92 | 2.07 | 1.71 | 2.49 |
| Belgium | 53 | 2.9 | 0.45 | 0.02 | 0.42 | 0.49 | 0.38 | 0.57 | -0.03 | 0.01 | -0.05 | 0.00 | -0.029 | -0.04 | 1.98 | 0.11 | 1.86 | 2.18 | 1.70 | 2.50 |
| Belize | 28 | 3.9 | 0.44 | 0.01 | 0.42 | 0.47 | 0.37 | 0.58 | -0.03 | 0.00 | -0.04 | -0.02 | -0.030 | -0.04 | 1.96 | 0.04 | 1.87 | 2.06 | 1.64 | 2.55 |
| Benin | 151 | 2.5 | 0.48 | 0.01 | 0.47 | 0.52 | 0.40 | 0.66 | 0.02 | 0.02 | -0.01 | 0.10 | 0.012 | 0.03 | 2.12 | 0.03 | 2.08 | 2.25 | 1.76 | 2.90 |
| Bhutan | 58 | 3.8 | 0.44 | 0.08 | 0.34 | 0.74 | 0.38 | 0.57 | 0.01 | 0.10 | -0.04 | 0.43 | 0.007 | 0.01 | 1.96 | 0.29 | 1.53 | 3.12 | 1.66 | 2.52 |
| Bolivia | 1242 | 2.6 | 0.47 | 0.04 | 0.36 | 0.72 | 0.39 | 0.63 | 0.00 | 0.06 | -0.05 | 0.37 | -0.001 | 0.01 | 2.07 | 0.15 | 1.61 | 3.03 | 1.73 | 2.77 |
| Bosnia and Herzegovina | 86 | 4.1 | 0.42 | 0.02 | 0.38 | 0.46 | 0.36 | 0.54 | -0.04 | 0.01 | -0.05 | -0.01 | -0.033 | -0.05 | 1.87 | 0.08 | 1.72 | 2.07 | 1.60 | 2.39 |
| Botswana | 716 | 1.2 | 0.57 | 0.08 | 0.45 | 0.79 | 0.47 | 0.76 | 0.17 | 0.11 | -0.05 | 0.48 | 0.133 | 0.25 | 2.42 | 0.29 | 1.99 | 3.34 | 2.03 | 3.25 |
| Brazil | 5287 | 3.2 | 0.46 | 0.04 | 0.34 | 0.79 | 0.39 | 0.59 | -0.01 | 0.05 | -0.05 | 0.50 | -0.006 | 0.00 | 2.02 | 0.14 | 1.55 | 3.38 | 1.70 | 2.60 |
| Brunei Darussalam | 8 | 4.0 | 0.45 | 0.02 | 0.41 | 0.47 | 0.36 | 0.60 | -0.04 | 0.01 | -0.04 | -0.03 | -0.029 | -0.05 | 1.98 | 0.09 | 1.84 | 2.09 | 1.62 | 2.66 |
| Bulgaria | 195 | 2.3 | 0.45 | 0.01 | 0.41 | 0.51 | 0.39 | 0.58 | -0.02 | 0.03 | -0.05 | 0.11 | -0.021 | -0.03 | 2.00 | 0.06 | 1.81 | 2.21 | 1.71 | 2.55 |
| Burkina Faso | 363 | 1.8 | 0.53 | 0.07 | 0.46 | 0.75 | 0.44 | 0.73 | 0.10 | 0.10 | -0.05 | 0.40 | 0.076 | 0.16 | 2.29 | 0.25 | 2.06 | 3.16 | 1.89 | 3.14 |
| Burundi | 34 | 3.3 | 0.45 | 0.01 | 0.43 | 0.47 | 0.38 | 0.59 | -0.02 | 0.01 | -0.04 | -0.01 | -0.021 | -0.03 | 2.00 | 0.03 | 1.93 | 2.06 | 1.69 | 2.58 |
| Cambodia | 233 | 3.3 | 0.46 | 0.02 | 0.41 | 0.50 | 0.38 | 0.61 | -0.02 | 0.02 | -0.04 | 0.03 | -0.018 | -0.03 | 2.03 | 0.06 | 1.84 | 2.18 | 1.69 | 2.69 |
| Cameroon | 558 | 3.0 | 0.46 | 0.02 | 0.43 | 0.70 | 0.38 | 0.60 | -0.02 | 0.03 | -0.05 | 0.35 | -0.019 | -0.03 | 2.02 | 0.07 | 1.91 | 2.94 | 1.70 | 2.62 |
| Canada | 3615 | 2.5 | 0.45 | 0.04 | 0.42 | 0.71 | 0.40 | 0.59 | 0.02 | 0.05 | -0.05 | 0.37 | 0.017 | 0.03 | 2.05 | 0.10 | 1.88 | 2.98 | 1.77 | 2.59 |
| Centr. African Rep. | 484 | 3.0 | 0.46 | 0.01 | 0.43 | 0.50 | 0.39 | 0.60 | -0.01 | 0.03 | -0.04 | 0.07 | -0.009 | -0.01 | 2.03 | 0.06 | 1.93 | 2.19 | 1.71 | 2.63 |
| Chad | 887 | 1.2 | 0.57 | 0.09 | 0.46 | 0.80 | 0.47 | 0.81 | 0.16 | 0.14 | -0.05 | 0.47 | 0.119 | 0.24 | 2.46 | 0.35 | 2.05 | 3.37 | 2.02 | 3.48 |
| Chile | 851 | 1.6 | 0.47 | 0.04 | 0.40 | 0.76 | 0.40 | 0.61 | 0.02 | 0.08 | -0.05 | 0.46 | 0.013 | 0.03 | 2.08 | 0.16 | 1.79 | 3.21 | 1.76 | 2.69 |
| China | 11951 | 2.5 | 0.47 | 0.07 | 0.30 | 0.76 | 0.40 | 0.62 | 0.03 | 0.10 | -0.05 | 0.45 | 0.019 | 0.04 | 2.07 | 0.25 | 1.36 | 3.19 | 1.76 | 2.73 |
| Colombia | 1210 | 3.5 | 0.46 | 0.03 | 0.36 | 0.80 | 0.38 | 0.61 | -0.03 | 0.03 | -0.05 | 0.48 | -0.022 | -0.03 | 2.02 | 0.10 | 1.64 | 3.39 | 1.67 | 2.68 |
| Comoros | 1 | 3.4 | 0.46 | 0.00 | 0.46 | 0.46 | 0.38 | 0.60 | -0.03 | 0.00 | -0.03 | -0.03 | -0.024 | -0.03 | 2.02 | 0.00 | 2.02 | 2.02 | 1.69 | 2.63 |
| Congo | 279 | 3.3 | 0.46 | 0.01 | 0.40 | 0.49 | 0.38 | 0.59 | -0.02 | 0.02 | -0.04 | 0.05 | -0.019 | -0.03 | 2.01 | 0.05 | 1.78 | 2.15 | 1.69 | 2.61 |
| Congo DRC | 1825 | 3.4 | 0.45 | 0.01 | 0.41 | 0.48 | 0.38 | 0.58 | -0.02 | 0.02 | -0.04 | 0.04 | -0.020 | -0.03 | 1.99 | 0.05 | 1.83 | 2.11 | 1.68 | 2.57 |
| Costa Rica | 56 | 3.7 | 0.46 | 0.01 | 0.43 | 0.50 | 0.37 | 0.62 | -0.03 | 0.02 | -0.04 | 0.02 | -0.025 | -0.04 | 2.02 | 0.06 | 1.90 | 2.22 | 1.66 | 2.73 |
| Côte d'Ivoire | 414 | 2.8 | 0.46 | 0.01 | 0.45 | 0.50 | 0.39 | 0.60 | -0.01 | 0.02 | -0.03 | 0.07 | -0.006 | 0.00 | 2.05 | 0.03 | 1.99 | 2.17 | 1.73 | 2.64 |
| Croatia | 100 | 3.8 | 0.42 | 0.03 | 0.33 | 0.49 | 0.36 | 0.54 | -0.04 | 0.01 | -0.05 | -0.02 | -0.033 | -0.05 | 1.88 | 0.13 | 1.51 | 2.18 | 1.61 | 2.39 |
| Cuba | 119 | 3.7 | 0.45 | 0.02 | 0.41 | 0.48 | 0.37 | 0.59 | -0.03 | 0.01 | -0.05 | 0.02 | -0.026 | -0.04 | 1.97 | 0.06 | 1.82 | 2.10 | 1.65 | 2.60 |
| Cyprus | 6 | 1.9 | 0.52 | 0.05 | 0.47 | 0.60 | 0.43 | 0.71 | 0.09 | 0.08 | 0.00 | 0.21 | 0.068 | 0.14 | 2.24 | 0.19 | 2.05 | 2.53 | 1.87 | 3.05 |
| Czech Republic | 161 | 3.1 | 0.44 | 0.02 | 0.41 | 0.49 | 0.38 | 0.56 | -0.04 | 0.01 | -0.05 | -0.01 | -0.031 | -0.04 | 1.96 | 0.11 | 1.82 | 2.17 | 1.68 | 2.46 |
| Denmark | 74 | 2.0 | 0.47 | 0.01 | 0.45 | 0.49 | 0.40 | 0.59 | 0.00 | 0.02 | -0.05 | 0.03 | -0.002 | 0.00 | 2.06 | 0.06 | 1.98 | 2.17 | 1.77 | 2.60 |
| Djibouti | 4 | 0.0 | 0.46 | 0.00 | 0.46 | 0.46 | 0.39 | 0.60 | -0.05 | 0.00 | -0.05 | -0.05 | -0.041 | -0.06 | 2.07 | 0.00 | 2.06 | 2.07 | 1.74 | 2.67 |
| Dominican Rep. | 54 | 4.1 | 0.44 | 0.02 | 0.38 | 0.48 | 0.36 | 0.58 | -0.04 | 0.01 | -0.04 | 0.02 | -0.030 | -0.05 | 1.94 | 0.08 | 1.70 | 2.10 | 1.62 | 2.58 |
| Ecuador | 264 | 3.1 | 0.47 | 0.05 | 0.37 | 0.75 | 0.39 | 0.61 | 0.01 | 0.07 | -0.05 | 0.40 | 0.006 | 0.02 | 2.05 | 0.19 | 1.68 | 3.13 | 1.72 | 2.68 |
| Egypt | 140 | 0.0 | 0.46 | 0.00 | 0.46 | 0.46 | 0.39 | 0.59 | -0.05 | 0.00 | -0.05 | -0.05 | -0.041 | -0.06 | 2.06 | 0.00 | 2.05 | 2.06 | 1.74 | 2.65 |
| El Salvador | 28 | 2.7 | 0.47 | 0.01 | 0.45 | 0.49 | 0.39 | 0.62 | 0.00 | 0.02 | -0.03 | 0.03 | 0.000 | 0.01 | 2.07 | 0.04 | 1.98 | 2.14 | 1.74 | 2.72 |
| Equatorial Guinea | 31 | 3.2 | 0.46 | 0.00 | 0.45 | 0.47 | 0.38 | 0.60 | -0.02 | 0.00 | -0.04 | -0.02 | -0.021 | -0.03 | 2.03 | 0.02 | 1.98 | 2.06 | 1.70 | 2.65 |
| Eritrea | 62 | 1.6 | 0.53 | 0.08 | 0.39 | 0.73 | 0.44 | 0.70 | 0.10 | 0.12 | -0.05 | 0.39 | 0.080 | 0.15 | 2.28 | 0.30 | 1.76 | 3.07 | 1.91 | 3.03 |
| Estonia | 98 | 2.2 | 0.48 | 0.04 | 0.45 | 0.66 | 0.41 | 0.60 | 0.04 | 0.05 | -0.01 | 0.24 | 0.029 | 0.05 | 2.08 | 0.16 | 1.99 | 2.78 | 1.80 | 2.63 |
| Ethiopia | 1286 | 2.2 | 0.51 | 0.09 | 0.34 | 0.79 | 0.41 | 0.71 | 0.05 | 0.13 | -0.05 | 0.46 | 0.035 | 0.08 | 2.21 | 0.34 | 1.57 | 3.34 | 1.82 | 3.11 |
| Faroe Islands | 1 | 1.8 | 0.50 | 0.00 | 0.50 | 0.50 | 0.42 | 0.63 | 0.08 | 0.00 | 0.08 | 0.08 | 0.065 | 0.11 | 2.16 | 0.00 | 2.16 | 2.16 | 1.85 | 2.74 |
| Fiji | 10 | 3.8 | 0.45 | 0.00 | 0.44 | 0.45 | 0.37 | 0.59 | -0.04 | 0.00 | -0.04 | -0.03 | -0.030 | -0.05 | 1.99 | 0.01 | 1.97 | 2.01 | 1.65 | 2.61 |
| Finland | 486 | 1.9 | 0.49 | 0.02 | 0.46 | 0.65 | 0.42 | 0.63 | 0.07 | 0.03 | 0.02 | 0.24 | 0.059 | 0.10 | 2.13 | 0.09 | 2.04 | 2.77 | 1.84 | 2.72 |
| France | 992 | 3.4 | 0.44 | 0.03 | 0.33 | 0.69 | 0.37 | 0.56 | -0.03 | 0.02 | -0.05 | 0.33 | -0.028 | -0.04 | 1.94 | 0.13 | 1.49 | 2.92 | 1.66 | 2.47 |
| French Guiana | 3 | 3.3 | 0.47 | 0.00 | 0.47 | 0.47 | 0.38 | 0.62 | -0.03 | 0.00 | -0.03 | -0.03 | -0.024 | -0.04 | 2.06 | 0.00 | 2.06 | 2.06 | 1.69 | 2.74 |
| Gabon | 251 | 3.5 | 0.45 | 0.02 | 0.39 | 0.48 | 0.38 | 0.58 | -0.02 | 0.01 | -0.04 | 0.02 | -0.021 | -0.03 | 1.99 | 0.09 | 1.76 | 2.11 | 1.67 | 2.58 |
| Gambia | 14 | 1.8 | 0.52 | 0.02 | 0.48 | 0.55 | 0.43 | 0.71 | 0.09 | 0.04 | 0.02 | 0.15 | 0.072 | 0.14 | 2.24 | 0.07 | 2.11 | 2.36 | 1.86 | 3.06 |
| Georgia | 106 | 4.2 | 0.42 | 0.07 | 0.29 | 0.72 | 0.36 | 0.54 | -0.01 | 0.08 | -0.04 | 0.38 | -0.012 | -0.01 | 1.88 | 0.26 | 1.35 | 3.02 | 1.61 | 2.41 |
| Germany | 680 | 2.7 | 0.45 | 0.02 | 0.41 | 0.49 | 0.39 | 0.57 | -0.03 | 0.02 | -0.05 | 0.04 | -0.023 | -0.03 | 2.00 | 0.09 | 1.84 | 2.18 | 1.72 | 2.52 |
| Ghana | 299 | 2.9 | 0.47 | 0.01 | 0.46 | 0.53 | 0.39 | 0.64 | -0.01 | 0.02 | -0.05 | 0.12 | -0.007 | 0.00 | 2.08 | 0.04 | 2.02 | 2.30 | 1.72 | 2.80 |
| Greece | 163 | 2.2 | 0.47 | 0.02 | 0.42 | 0.52 | 0.39 | 0.62 | -0.01 | 0.04 | -0.05 | 0.10 | -0.008 | -0.01 | 2.05 | 0.07 | 1.88 | 2.24 | 1.73 | 2.72 |
| Guadeloupe | 1 | 3.6 | 0.46 | 0.00 | 0.46 | 0.46 | 0.38 | 0.60 | -0.03 | 0.00 | -0.03 | -0.03 | -0.027 | -0.04 | 2.02 | 0.00 | 2.02 | 2.02 | 1.68 | 2.64 |
| Guatemala | 136 | 3.6 | 0.45 | 0.02 | 0.34 | 0.49 | 0.37 | 0.59 | -0.03 | 0.01 | -0.04 | 0.03 | -0.024 | -0.03 | 1.98 | 0.07 | 1.57 | 2.15 | 1.66 | 2.58 |
| Guinea | 319 | 2.7 | 0.48 | 0.01 | 0.44 | 0.53 | 0.39 | 0.64 | 0.00 | 0.03 | -0.04 | 0.12 | 0.000 | 0.01 | 2.09 | 0.05 | 1.97 | 2.29 | 1.74 | 2.81 |
| Guinea-Bissau | 38 | 2.3 | 0.49 | 0.01 | 0.48 | 0.54 | 0.40 | 0.69 | 0.03 | 0.02 | 0.02 | 0.12 | 0.020 | 0.05 | 2.17 | 0.03 | 2.13 | 2.32 | 1.77 | 3.03 |
| Guyana | 160 | 3.3 | 0.46 | 0.01 | 0.45 | 0.51 | 0.38 | 0.61 | -0.02 | 0.02 | -0.05 | 0.09 | -0.021 | -0.03 | 2.03 | 0.03 | 1.98 | 2.23 | 1.69 | 2.67 |
| Haiti | 29 | 3.1 | 0.45 | 0.02 | 0.41 | 0.47 | 0.37 | 0.59 | -0.04 | 0.01 | -0.05 | -0.01 | -0.032 | -0.05 | 1.99 | 0.07 | 1.84 | 2.09 | 1.66 | 2.64 |
| Honduras | 136 | 3.6 | 0.45 | 0.01 | 0.41 | 0.48 | 0.38 | 0.59 | -0.03 | 0.01 | -0.04 | 0.00 | -0.025 | -0.04 | 1.99 | 0.05 | 1.84 | 2.10 | 1.67 | 2.62 |
| Hungary | 159 | 2.1 | 0.46 | 0.03 | 0.40 | 0.52 | 0.40 | 0.59 | -0.02 | 0.04 | -0.05 | 0.12 | -0.014 | -0.02 | 2.05 | 0.12 | 1.78 | 2.23 | 1.75 | 2.60 |
| Iceland | 57 | 0.5 | 0.62 | 0.06 | 0.51 | 0.66 | 0.51 | 0.85 | 0.21 | 0.05 | 0.09 | 0.24 | 0.161 | 0.30 | 2.64 | 0.22 | 2.23 | 2.81 | 2.19 | 3.61 |
| India | 4243 | 2.5 | 0.49 | 0.06 | 0.27 | 0.80 | 0.41 | 0.66 | 0.04 | 0.09 | -0.05 | 0.47 | 0.030 | 0.06 | 2.15 | 0.24 | 1.26 | 3.37 | 1.78 | 2.90 |
| Indonesia | 1829 | 3.6 | 0.46 | 0.02 | 0.38 | 0.51 | 0.38 | 0.61 | -0.03 | 0.01 | -0.05 | 0.09 | -0.025 | -0.04 | 2.03 | 0.06 | 1.70 | 2.24 | 1.67 | 2.71 |
| Iran | 1200 | 0.5 | 0.50 | 0.06 | 0.40 | 0.80 | 0.42 | 0.66 | 0.03 | 0.12 | -0.05 | 0.48 | 0.024 | 0.05 | 2.18 | 0.23 | 1.79 | 3.39 | 1.84 | 2.86 |
| Iraq | 273 | 0.2 | 0.47 | 0.05 | 0.46 | 0.75 | 0.40 | 0.62 | -0.02 | 0.08 | -0.05 | 0.40 | -0.020 | -0.02 | 2.10 | 0.17 | 2.02 | 3.13 | 1.77 | 2.73 |
| Ireland | 101 | 2.1 | 0.47 | 0.02 | 0.43 | 0.49 | 0.39 | 0.60 | -0.04 | 0.01 | -0.05 | 0.00 | -0.031 | -0.04 | 2.06 | 0.10 | 1.91 | 2.19 | 1.75 | 2.66 |
| Israel | 17 | 0.9 | 0.51 | 0.06 | 0.46 | 0.68 | 0.43 | 0.69 | 0.08 | 0.10 | -0.05 | 0.32 | 0.059 | 0.12 | 2.23 | 0.21 | 2.06 | 2.88 | 1.87 | 2.99 |
| Italy | 468 | 2.8 | 0.45 | 0.04 | 0.34 | 0.72 | 0.38 | 0.58 | -0.02 | 0.05 | -0.05 | 0.39 | -0.017 | -0.02 | 1.99 | 0.15 | 1.55 | 3.04 | 1.69 | 2.54 |
| Jamaica | 12 | 4.3 | 0.43 | 0.01 | 0.41 | 0.44 | 0.36 | 0.55 | -0.04 | 0.00 | -0.04 | -0.03 | -0.033 | -0.05 | 1.90 | 0.05 | 1.84 | 1.97 | 1.60 | 2.46 |
| Japan | 502 | 4.4 | 0.41 | 0.03 | 0.34 | 0.53 | 0.35 | 0.53 | -0.04 | 0.01 | -0.04 | 0.13 | -0.030 | -0.04 | 1.84 | 0.12 | 1.55 | 2.27 | 1.57 | 2.36 |
| Jordan | 36 | 0.2 | 0.50 | 0.06 | 0.46 | 0.68 | 0.42 | 0.68 | 0.04 | 0.12 | -0.05 | 0.31 | 0.028 | 0.07 | 2.20 | 0.23 | 2.05 | 2.85 | 1.85 | 2.95 |
| Kazakhstan | 3776 | 0.4 | 0.56 | 0.10 | 0.43 | 0.78 | 0.48 | 0.74 | 0.14 | 0.17 | -0.05 | 0.46 | 0.109 | 0.20 | 2.41 | 0.37 | 1.92 | 3.31 | 2.05 | 3.16 |
| Kenya | 566 | 1.8 | 0.53 | 0.12 | 0.37 | 0.81 | 0.45 | 0.71 | 0.10 | 0.17 | -0.05 | 0.49 | 0.078 | 0.16 | 2.31 | 0.44 | 1.67 | 3.43 | 1.94 | 3.06 |
| Kyrgyzstan | 311 | 1.9 | 0.50 | 0.07 | 0.41 | 0.75 | 0.43 | 0.67 | 0.08 | 0.11 | -0.05 | 0.44 | 0.061 | 0.11 | 2.19 | 0.25 | 1.84 | 3.18 | 1.87 | 2.89 |
| Laos | 314 | 3.8 | 0.45 | 0.02 | 0.42 | 0.50 | 0.37 | 0.61 | -0.03 | 0.01 | -0.04 | 0.03 | -0.027 | -0.04 | 1.99 | 0.06 | 1.89 | 2.17 | 1.65 | 2.69 |
| Latvia | 141 | 2.6 | 0.46 | 0.01 | 0.44 | 0.49 | 0.39 | 0.58 | 0.00 | 0.02 | -0.05 | 0.04 | 0.001 | 0.01 | 2.01 | 0.03 | 1.95 | 2.17 | 1.74 | 2.53 |
| Lebanon | 16 | 1.9 | 0.49 | 0.03 | 0.46 | 0.55 | 0.40 | 0.66 | 0.02 | 0.06 | -0.05 | 0.15 | 0.016 | 0.04 | 2.13 | 0.10 | 2.02 | 2.36 | 1.78 | 2.89 |
| Lesotho | 45 | 3.2 | 0.43 | 0.03 | 0.33 | 0.47 | 0.37 | 0.57 | -0.03 | 0.02 | -0.05 | 0.02 | -0.029 | -0.04 | 1.93 | 0.13 | 1.50 | 2.07 | 1.64 | 2.51 |
| Liberia | 114 | 3.4 | 0.46 | 0.01 | 0.44 | 0.48 | 0.38 | 0.61 | -0.03 | 0.01 | -0.04 | 0.00 | -0.023 | -0.03 | 2.04 | 0.03 | 1.97 | 2.11 | 1.69 | 2.69 |
| Libya | 65 | 0.3 | 0.53 | 0.11 | 0.46 | 0.78 | 0.44 | 0.72 | 0.08 | 0.17 | -0.05 | 0.44 | 0.058 | 0.13 | 2.31 | 0.40 | 2.05 | 3.26 | 1.93 | 3.10 |
| Liechtenstein | 1 | 1.8 | 0.50 | 0.00 | 0.50 | 0.50 | 0.43 | 0.65 | 0.09 | 0.00 | 0.09 | 0.09 | 0.074 | 0.13 | 2.18 | 0.00 | 2.18 | 2.18 | 1.86 | 2.80 |
| Lithuania | 127 | 2.6 | 0.46 | 0.01 | 0.44 | 0.49 | 0.39 | 0.57 | -0.01 | 0.02 | -0.05 | 0.02 | -0.008 | -0.01 | 2.01 | 0.05 | 1.96 | 2.17 | 1.74 | 2.52 |
| Luxembourg | 5 | 3.8 | 0.43 | 0.01 | 0.42 | 0.44 | 0.37 | 0.54 | -0.03 | 0.00 | -0.04 | -0.03 | -0.030 | -0.04 | 1.91 | 0.03 | 1.87 | 1.97 | 1.65 | 2.40 |
| Madagascar | 760 | 2.7 | 0.48 | 0.04 | 0.38 | 0.75 | 0.40 | 0.62 | 0.01 | 0.07 | -0.05 | 0.41 | 0.010 | 0.02 | 2.09 | 0.17 | 1.72 | 3.15 | 1.76 | 2.71 |
| Malawi | 123 | 2.4 | 0.48 | 0.01 | 0.46 | 0.52 | 0.40 | 0.62 | 0.02 | 0.03 | -0.02 | 0.11 | 0.014 | 0.03 | 2.09 | 0.05 | 2.02 | 2.26 | 1.76 | 2.73 |
| Malaysia | 393 | 4.0 | 0.44 | 0.02 | 0.39 | 0.48 | 0.36 | 0.59 | -0.04 | 0.01 | -0.04 | -0.02 | -0.031 | -0.05 | 1.96 | 0.06 | 1.74 | 2.10 | 1.62 | 2.60 |
| Mali | 794 | 1.2 | 0.56 | 0.10 | 0.46 | 0.80 | 0.46 | 0.80 | 0.13 | 0.15 | -0.05 | 0.48 | 0.098 | 0.21 | 2.42 | 0.37 | 2.07 | 3.38 | 1.97 | 3.44 |
| Mauritania | 303 | 0.2 | 0.69 | 0.13 | 0.46 | 0.81 | 0.56 | 1.00 | 0.31 | 0.20 | -0.05 | 0.49 | 0.232 | 0.50 | 2.93 | 0.51 | 2.06 | 3.44 | 2.37 | 4.24 |
| Mexico | 2592 | 2.4 | 0.49 | 0.08 | 0.33 | 0.81 | 0.41 | 0.67 | 0.04 | 0.13 | -0.05 | 0.50 | 0.026 | 0.06 | 2.16 | 0.32 | 1.51 | 3.43 | 1.80 | 2.92 |
| Moldova | 69 | 2.9 | 0.46 | 0.02 | 0.42 | 0.51 | 0.39 | 0.58 | 0.01 | 0.04 | -0.04 | 0.11 | 0.003 | 0.01 | 2.01 | 0.09 | 1.86 | 2.22 | 1.73 | 2.57 |
| Mongolia | 1821 | 0.6 | 0.53 | 0.05 | 0.44 | 0.74 | 0.46 | 0.68 | 0.12 | 0.11 | -0.05 | 0.42 | 0.100 | 0.16 | 2.30 | 0.18 | 1.96 | 3.11 | 1.99 | 2.92 |
| Montenegro | 23 | 4.1 | 0.41 | 0.03 | 0.36 | 0.46 | 0.35 | 0.53 | -0.04 | 0.01 | -0.05 | -0.02 | -0.032 | -0.05 | 1.85 | 0.13 | 1.62 | 2.06 | 1.58 | 2.37 |
| Morocco | 359 | 1.5 | 0.49 | 0.06 | 0.43 | 0.77 | 0.41 | 0.65 | 0.02 | 0.09 | -0.05 | 0.43 | 0.014 | 0.04 | 2.14 | 0.21 | 1.89 | 3.23 | 1.79 | 2.86 |
| Mozambique | 1016 | 2.3 | 0.49 | 0.03 | 0.41 | 0.67 | 0.41 | 0.67 | 0.04 | 0.06 | -0.05 | 0.31 | 0.025 | 0.06 | 2.15 | 0.12 | 1.83 | 2.82 | 1.78 | 2.93 |
| Myanmar | 855 | 3.6 | 0.46 | 0.03 | 0.33 | 0.51 | 0.37 | 0.64 | -0.03 | 0.01 | -0.05 | 0.07 | -0.023 | -0.03 | 2.03 | 0.11 | 1.49 | 2.25 | 1.66 | 2.81 |
| Namibia | 839 | 1.0 | 0.55 | 0.08 | 0.46 | 0.79 | 0.46 | 0.77 | 0.14 | 0.12 | -0.05 | 0.47 | 0.107 | 0.22 | 2.39 | 0.30 | 2.03 | 3.34 | 1.98 | 3.31 |
| Nepal | 217 | 3.8 | 0.43 | 0.08 | 0.30 | 0.77 | 0.36 | 0.56 | -0.01 | 0.08 | -0.05 | 0.45 | -0.006 | 0.00 | 1.91 | 0.30 | 1.36 | 3.26 | 1.62 | 2.47 |
| Netherlands | 51 | 2.3 | 0.46 | 0.02 | 0.42 | 0.49 | 0.39 | 0.58 | -0.03 | 0.02 | -0.05 | 0.00 | -0.023 | -0.03 | 2.03 | 0.09 | 1.87 | 2.17 | 1.74 | 2.57 |
| New Caledonia | 14 | 3.8 | 0.45 | 0.01 | 0.43 | 0.46 | 0.37 | 0.60 | -0.03 | 0.00 | -0.04 | -0.03 | -0.029 | -0.04 | 1.99 | 0.02 | 1.93 | 2.02 | 1.66 | 2.64 |
| New Zealand | 354 | 4.2 | 0.42 | 0.03 | 0.33 | 0.51 | 0.36 | 0.53 | -0.03 | 0.02 | -0.05 | 0.09 | -0.028 | -0.04 | 1.87 | 0.12 | 1.50 | 2.21 | 1.60 | 2.38 |
| Nicaragua | 152 | 3.2 | 0.46 | 0.01 | 0.44 | 0.49 | 0.38 | 0.61 | -0.02 | 0.02 | -0.04 | 0.04 | -0.018 | -0.03 | 2.04 | 0.04 | 1.95 | 2.15 | 1.70 | 2.70 |
| Niger | 586 | 0.4 | 0.61 | 0.12 | 0.46 | 0.80 | 0.50 | 0.89 | 0.20 | 0.18 | -0.05 | 0.47 | 0.149 | 0.33 | 2.62 | 0.44 | 2.06 | 3.38 | 2.13 | 3.76 |
| Nigeria | 1163 | 2.2 | 0.48 | 0.04 | 0.45 | 0.78 | 0.40 | 0.63 | 0.02 | 0.07 | -0.05 | 0.45 | 0.011 | 0.03 | 2.11 | 0.16 | 1.98 | 3.30 | 1.77 | 2.77 |
| North Korea | 188 | 3.7 | 0.44 | 0.03 | 0.39 | 0.54 | 0.37 | 0.58 | -0.02 | 0.04 | -0.04 | 0.13 | -0.016 | -0.02 | 1.96 | 0.11 | 1.74 | 2.31 | 1.66 | 2.58 |
| Norway | 397 | 1.8 | 0.52 | 0.08 | 0.44 | 0.81 | 0.44 | 0.69 | 0.08 | 0.10 | -0.03 | 0.50 | 0.064 | 0.12 | 2.25 | 0.30 | 1.96 | 3.44 | 1.90 | 2.96 |
| Pakistan | 969 | 0.8 | 0.54 | 0.13 | 0.27 | 0.81 | 0.44 | 0.73 | 0.11 | 0.18 | -0.05 | 0.49 | 0.080 | 0.17 | 2.32 | 0.50 | 1.25 | 3.42 | 1.94 | 3.15 |
| Palestinian Ter. | 9 | 0.8 | 0.53 | 0.04 | 0.46 | 0.56 | 0.44 | 0.70 | 0.11 | 0.09 | -0.05 | 0.17 | 0.084 | 0.15 | 2.28 | 0.13 | 2.06 | 2.39 | 1.92 | 2.99 |
| Panama | 82 | 3.4 | 0.46 | 0.01 | 0.43 | 0.54 | 0.38 | 0.61 | -0.02 | 0.02 | -0.04 | 0.10 | -0.021 | -0.03 | 2.03 | 0.05 | 1.93 | 2.35 | 1.69 | 2.67 |
| Papua New Guinea | 142 | 3.7 | 0.46 | 0.02 | 0.38 | 0.48 | 0.37 | 0.61 | -0.03 | 0.01 | -0.04 | 0.02 | -0.026 | -0.04 | 2.02 | 0.07 | 1.72 | 2.11 | 1.66 | 2.70 |
| Paraguay | 487 | 3.3 | 0.47 | 0.06 | 0.36 | 0.73 | 0.39 | 0.61 | 0.01 | 0.09 | -0.04 | 0.37 | 0.007 | 0.02 | 2.05 | 0.24 | 1.63 | 3.05 | 1.72 | 2.67 |
| Peru | 941 | 1.8 | 0.48 | 0.06 | 0.35 | 0.81 | 0.40 | 0.63 | 0.01 | 0.10 | -0.05 | 0.50 | 0.003 | 0.02 | 2.10 | 0.23 | 1.60 | 3.43 | 1.76 | 2.75 |
| Philippines | 284 | 3.9 | 0.44 | 0.02 | 0.40 | 0.48 | 0.37 | 0.59 | -0.03 | 0.01 | -0.05 | 0.00 | -0.029 | -0.04 | 1.97 | 0.08 | 1.78 | 2.10 | 1.64 | 2.59 |
| Poland | 595 | 2.4 | 0.46 | 0.02 | 0.40 | 0.49 | 0.39 | 0.57 | -0.02 | 0.02 | -0.05 | 0.06 | -0.022 | -0.03 | 2.01 | 0.09 | 1.80 | 2.17 | 1.73 | 2.53 |
| Portugal | 135 | 3.5 | 0.44 | 0.05 | 0.31 | 0.51 | 0.37 | 0.59 | -0.02 | 0.03 | -0.05 | 0.09 | -0.017 | -0.02 | 1.95 | 0.19 | 1.42 | 2.22 | 1.64 | 2.59 |
| Puerto Rico | 11 | 3.8 | 0.46 | 0.01 | 0.42 | 0.46 | 0.37 | 0.63 | -0.03 | 0.00 | -0.04 | -0.03 | -0.029 | -0.05 | 2.02 | 0.04 | 1.89 | 2.05 | 1.65 | 2.80 |
| Réunion | 1 | 3.6 | 0.45 | 0.00 | 0.45 | 0.45 | 0.38 | 0.57 | -0.03 | 0.00 | -0.03 | -0.03 | -0.027 | -0.04 | 1.98 | 0.00 | 1.98 | 1.98 | 1.68 | 2.53 |
| Romania | 439 | 2.0 | 0.46 | 0.03 | 0.38 | 0.61 | 0.39 | 0.58 | -0.03 | 0.03 | -0.05 | 0.25 | -0.027 | -0.04 | 2.03 | 0.11 | 1.72 | 2.59 | 1.73 | 2.57 |
| Russian Federation | 18021 | 1.7 | 0.52 | 0.06 | 0.35 | 0.80 | 0.45 | 0.67 | 0.11 | 0.10 | -0.05 | 0.49 | 0.092 | 0.15 | 2.24 | 0.23 | 1.57 | 3.40 | 1.93 | 2.87 |
| Rwanda | 30 | 3.7 | 0.44 | 0.01 | 0.41 | 0.46 | 0.37 | 0.56 | -0.03 | 0.01 | -0.04 | -0.02 | -0.027 | -0.04 | 1.95 | 0.05 | 1.82 | 2.04 | 1.66 | 2.49 |
| Sao Tome and Principe | 2 | 2.9 | 0.47 | 0.00 | 0.46 | 0.47 | 0.39 | 0.61 | -0.01 | 0.01 | -0.02 | -0.01 | -0.014 | -0.02 | 2.05 | 0.00 | 2.05 | 2.06 | 1.72 | 2.67 |
| Saudi Arabia | 392 | 0.0 | 0.47 | 0.06 | 0.46 | 0.79 | 0.40 | 0.62 | -0.03 | 0.09 | -0.05 | 0.46 | -0.028 | -0.04 | 2.10 | 0.22 | 2.05 | 3.31 | 1.77 | 2.74 |
| Senegal | 220 | 1.5 | 0.56 | 0.09 | 0.48 | 0.81 | 0.46 | 0.79 | 0.16 | 0.12 | 0.02 | 0.48 | 0.118 | 0.24 | 2.43 | 0.35 | 2.10 | 3.40 | 1.99 | 3.40 |
| Serbia | 144 | 3.3 | 0.44 | 0.02 | 0.38 | 0.49 | 0.38 | 0.56 | -0.04 | 0.01 | -0.05 | 0.03 | -0.031 | -0.04 | 1.95 | 0.10 | 1.73 | 2.17 | 1.67 | 2.47 |
| Sierra Leone | 90 | 3.2 | 0.48 | 0.01 | 0.46 | 0.50 | 0.38 | 0.67 | -0.02 | 0.01 | -0.03 | 0.01 | -0.019 | -0.03 | 2.11 | 0.04 | 2.04 | 2.18 | 1.70 | 2.96 |
| Slovakia | 99 | 2.7 | 0.45 | 0.03 | 0.42 | 0.49 | 0.39 | 0.57 | -0.04 | 0.01 | -0.05 | 0.02 | -0.031 | -0.04 | 1.99 | 0.12 | 1.85 | 2.17 | 1.71 | 2.51 |
| Slovenia | 36 | 4.1 | 0.42 | 0.02 | 0.38 | 0.49 | 0.36 | 0.54 | -0.04 | 0.01 | -0.05 | -0.02 | -0.032 | -0.05 | 1.88 | 0.08 | 1.71 | 2.18 | 1.61 | 2.38 |
| Solomon Islands | 17 | 3.8 | 0.46 | 0.02 | 0.42 | 0.48 | 0.37 | 0.62 | -0.03 | 0.00 | -0.04 | -0.03 | -0.028 | -0.05 | 2.02 | 0.07 | 1.87 | 2.10 | 1.65 | 2.74 |
| Somalia | 644 | 0.3 | 0.64 | 0.11 | 0.46 | 0.81 | 0.53 | 0.91 | 0.26 | 0.17 | -0.05 | 0.50 | 0.194 | 0.40 | 2.74 | 0.43 | 2.04 | 3.42 | 2.24 | 3.85 |
| South Africa | 1622 | 2.3 | 0.49 | 0.10 | 0.32 | 0.83 | 0.41 | 0.65 | 0.04 | 0.15 | -0.05 | 0.53 | 0.032 | 0.07 | 2.13 | 0.40 | 1.46 | 3.53 | 1.80 | 2.84 |
| South Korea | 144 | 4.6 | 0.41 | 0.02 | 0.37 | 0.45 | 0.35 | 0.54 | -0.04 | 0.00 | -0.04 | -0.03 | -0.034 | -0.05 | 1.84 | 0.09 | 1.67 | 1.98 | 1.56 | 2.40 |
| South Sudan | 467 | 2.3 | 0.50 | 0.04 | 0.46 | 0.77 | 0.41 | 0.74 | 0.04 | 0.06 | -0.04 | 0.46 | 0.028 | 0.07 | 2.20 | 0.14 | 2.03 | 3.25 | 1.79 | 3.21 |
| Spain | 807 | 2.4 | 0.45 | 0.03 | 0.31 | 0.69 | 0.38 | 0.60 | -0.02 | 0.04 | -0.05 | 0.33 | -0.018 | -0.02 | 2.01 | 0.11 | 1.44 | 2.92 | 1.70 | 2.65 |
| Sri Lanka | 78 | 2.9 | 0.49 | 0.02 | 0.43 | 0.54 | 0.39 | 0.71 | 0.00 | 0.03 | -0.05 | 0.10 | -0.005 | 0.00 | 2.15 | 0.07 | 1.93 | 2.33 | 1.72 | 3.13 |
| Sudan | 769 | 1.3 | 0.57 | 0.08 | 0.46 | 0.81 | 0.46 | 0.85 | 0.16 | 0.12 | -0.05 | 0.49 | 0.117 | 0.25 | 2.47 | 0.30 | 2.06 | 3.41 | 2.00 | 3.61 |
| Suriname | 20 | 3.6 | 0.45 | 0.01 | 0.44 | 0.46 | 0.38 | 0.59 | -0.03 | 0.00 | -0.04 | -0.02 | -0.027 | -0.04 | 2.00 | 0.03 | 1.96 | 2.05 | 1.67 | 2.61 |
| Swaziland | 25 | 3.5 | 0.46 | 0.04 | 0.41 | 0.59 | 0.38 | 0.63 | -0.01 | 0.06 | -0.04 | 0.20 | -0.010 | -0.01 | 2.02 | 0.16 | 1.83 | 2.52 | 1.68 | 2.77 |
| Sweden | 543 | 2.1 | 0.48 | 0.04 | 0.45 | 0.70 | 0.42 | 0.62 | 0.05 | 0.06 | -0.05 | 0.35 | 0.042 | 0.07 | 2.11 | 0.14 | 1.98 | 2.95 | 1.82 | 2.70 |
| Switzerland | 75 | 2.7 | 0.46 | 0.05 | 0.39 | 0.71 | 0.39 | 0.59 | -0.01 | 0.07 | -0.05 | 0.35 | -0.010 | -0.01 | 2.02 | 0.19 | 1.77 | 2.97 | 1.73 | 2.59 |
| Syria | 198 | 0.4 | 0.49 | 0.05 | 0.45 | 0.72 | 0.41 | 0.64 | 0.03 | 0.09 | -0.05 | 0.37 | 0.018 | 0.04 | 2.15 | 0.16 | 2.00 | 3.02 | 1.81 | 2.82 |
| Tajikistan | 195 | 1.8 | 0.52 | 0.08 | 0.44 | 0.74 | 0.44 | 0.71 | 0.08 | 0.13 | -0.05 | 0.43 | 0.065 | 0.12 | 2.25 | 0.29 | 1.94 | 3.14 | 1.89 | 3.07 |
| Tanzania | 1130 | 2.5 | 0.48 | 0.02 | 0.41 | 0.73 | 0.40 | 0.66 | 0.02 | 0.04 | -0.05 | 0.37 | 0.013 | 0.03 | 2.11 | 0.09 | 1.85 | 3.05 | 1.76 | 2.87 |
| Thailand | 664 | 3.4 | 0.46 | 0.02 | 0.41 | 0.51 | 0.38 | 0.65 | -0.02 | 0.02 | -0.04 | 0.04 | -0.020 | -0.03 | 2.05 | 0.07 | 1.86 | 2.23 | 1.68 | 2.85 |
| Macedonia | 39 | 2.9 | 0.45 | 0.02 | 0.41 | 0.50 | 0.38 | 0.59 | -0.02 | 0.03 | -0.05 | 0.08 | -0.014 | -0.02 | 2.00 | 0.07 | 1.84 | 2.17 | 1.70 | 2.59 |
| Timor-Leste | 16 | 3.0 | 0.46 | 0.01 | 0.44 | 0.49 | 0.39 | 0.61 | -0.01 | 0.02 | -0.04 | 0.05 | -0.009 | -0.01 | 2.05 | 0.04 | 1.95 | 2.13 | 1.72 | 2.66 |
| Togo | 78 | 2.3 | 0.47 | 0.01 | 0.45 | 0.48 | 0.39 | 0.62 | -0.01 | 0.02 | -0.05 | 0.03 | -0.010 | -0.01 | 2.07 | 0.02 | 2.00 | 2.11 | 1.73 | 2.71 |
| Trinidad & Tobago | 6 | 3.7 | 0.45 | 0.01 | 0.44 | 0.46 | 0.37 | 0.60 | -0.03 | 0.00 | -0.04 | -0.03 | -0.028 | -0.04 | 2.00 | 0.03 | 1.96 | 2.05 | 1.66 | 2.66 |
| Tunisia | 93 | 1.0 | 0.50 | 0.07 | 0.45 | 0.78 | 0.41 | 0.67 | 0.03 | 0.11 | -0.05 | 0.48 | 0.020 | 0.05 | 2.18 | 0.25 | 1.98 | 3.31 | 1.82 | 2.93 |
| Turkey | 1219 | 1.2 | 0.46 | 0.02 | 0.31 | 0.56 | 0.39 | 0.59 | -0.02 | 0.04 | -0.05 | 0.17 | -0.022 | -0.03 | 2.04 | 0.08 | 1.41 | 2.38 | 1.73 | 2.62 |
| Turkmenistan | 496 | 0.1 | 0.49 | 0.06 | 0.46 | 0.71 | 0.41 | 0.64 | 0.00 | 0.11 | -0.05 | 0.38 | 0.001 | 0.01 | 2.15 | 0.22 | 2.05 | 2.98 | 1.81 | 2.82 |
| Uganda | 245 | 3.6 | 0.45 | 0.02 | 0.39 | 0.53 | 0.37 | 0.61 | -0.03 | 0.02 | -0.05 | 0.12 | -0.025 | -0.04 | 1.99 | 0.06 | 1.77 | 2.28 | 1.66 | 2.68 |
| Ukraine | 1134 | 2.9 | 0.46 | 0.03 | 0.41 | 0.62 | 0.39 | 0.58 | 0.01 | 0.06 | -0.05 | 0.25 | 0.004 | 0.01 | 2.01 | 0.12 | 1.81 | 2.60 | 1.74 | 2.53 |
| United Kingdom | 401 | 2.2 | 0.47 | 0.05 | 0.42 | 0.67 | 0.40 | 0.61 | -0.01 | 0.06 | -0.05 | 0.24 | -0.010 | -0.01 | 2.08 | 0.18 | 1.87 | 2.82 | 1.77 | 2.67 |
| United States | 11910 | 3.0 | 0.46 | 0.08 | 0.33 | 0.83 | 0.40 | 0.60 | 0.02 | 0.11 | -0.05 | 0.53 | 0.016 | 0.04 | 2.04 | 0.30 | 1.52 | 3.55 | 1.74 | 2.61 |
| Uruguay | 252 | 4.8 | 0.41 | 0.03 | 0.35 | 0.46 | 0.34 | 0.54 | -0.04 | 0.00 | -0.04 | -0.03 | -0.034 | -0.05 | 1.82 | 0.11 | 1.58 | 2.02 | 1.53 | 2.41 |
| Uzbekistan | 506 | 0.4 | 0.51 | 0.07 | 0.45 | 0.71 | 0.42 | 0.69 | 0.05 | 0.13 | -0.05 | 0.38 | 0.034 | 0.08 | 2.22 | 0.26 | 2.01 | 2.99 | 1.86 | 3.01 |
| Vanuatu | 1 | 3.7 | 0.45 | 0.00 | 0.45 | 0.45 | 0.37 | 0.60 | -0.03 | 0.00 | -0.03 | -0.03 | -0.029 | -0.04 | 2.01 | 0.00 | 2.01 | 2.01 | 1.66 | 2.64 |
| Venezuela | 730 | 3.0 | 0.46 | 0.03 | 0.39 | 0.72 | 0.39 | 0.61 | -0.01 | 0.04 | -0.05 | 0.35 | -0.009 | -0.01 | 2.04 | 0.10 | 1.76 | 3.01 | 1.71 | 2.66 |
| Vietnam | 426 | 3.7 | 0.45 | 0.02 | 0.39 | 0.52 | 0.37 | 0.61 | -0.03 | 0.02 | -0.04 | 0.10 | -0.024 | -0.04 | 2.00 | 0.08 | 1.75 | 2.25 | 1.66 | 2.70 |
| Yemen | 198 | 0.2 | 0.54 | 0.11 | 0.46 | 0.78 | 0.45 | 0.74 | 0.09 | 0.17 | -0.05 | 0.44 | 0.065 | 0.14 | 2.34 | 0.40 | 2.05 | 3.27 | 1.94 | 3.21 |
| Zambia | 991 | 2.5 | 0.48 | 0.01 | 0.44 | 0.53 | 0.40 | 0.64 | 0.01 | 0.02 | -0.05 | 0.13 | 0.008 | 0.02 | 2.09 | 0.04 | 1.96 | 2.29 | 1.75 | 2.78 |
| Zimbabwe | 535 | 2.3 | 0.49 | 0.04 | 0.42 | 0.79 | 0.41 | 0.67 | 0.05 | 0.07 | -0.04 | 0.48 | 0.035 | 0.07 | 2.15 | 0.15 | 1.87 | 3.36 | 1.80 | 2.90 |

| 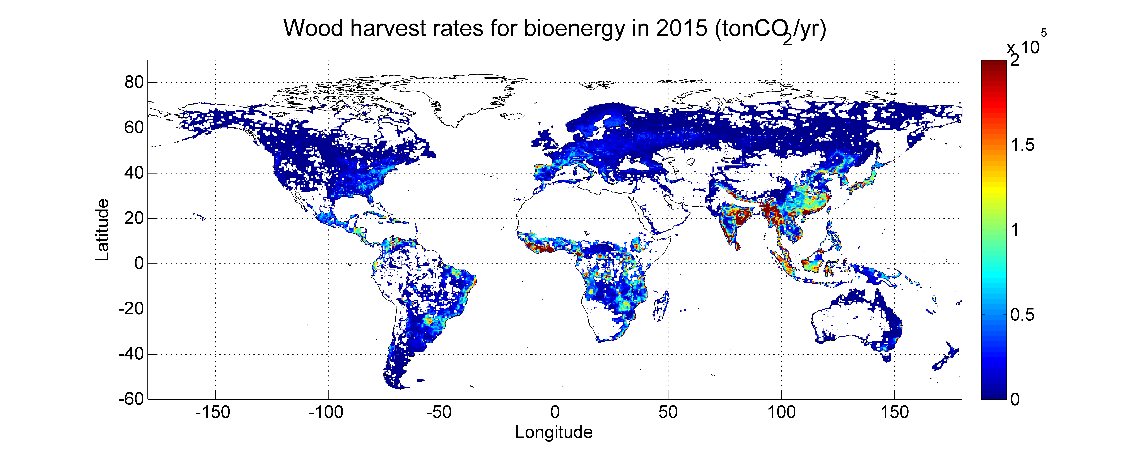  a) |
| --- |
| 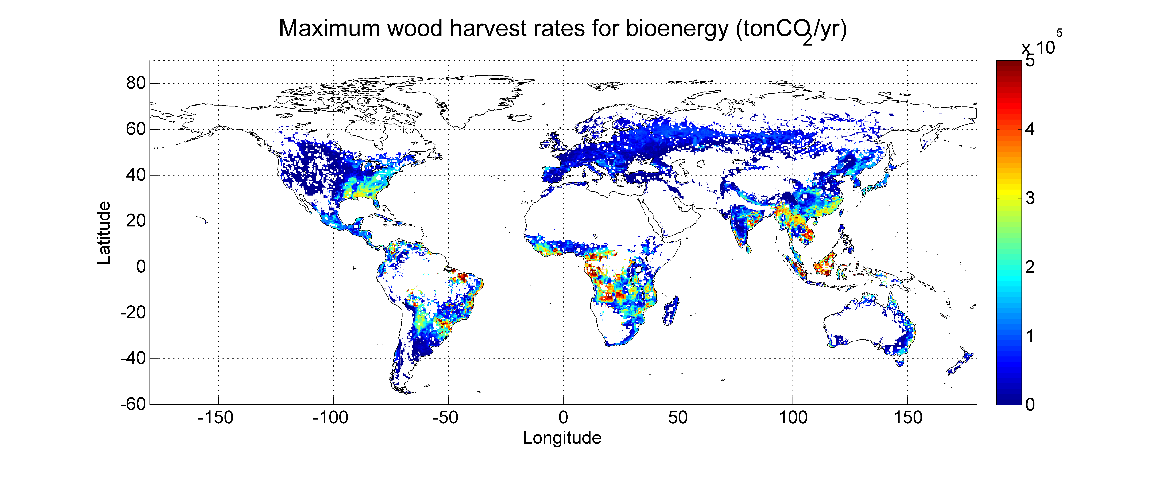  b) |
| 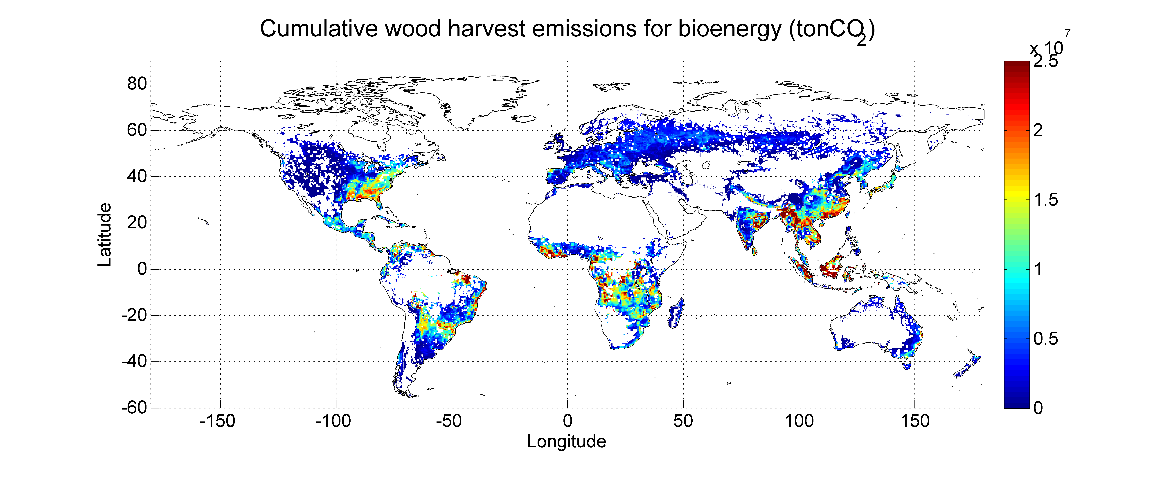  c) |

Figure S5 Spatially distributed CO_2_ emissions from wood harvest bioenergy production in the RCP8.5 scenario. (a) shows the CO_2_ emissions from forest bioenergy in 2015, (b) shows the maximum wood harvest rates in each grid cell across the emission scenario (i.e., from 2015 to 2100), and (c) shows the cumulative emissions in each grid cell up to 2100. Maps are generated using the software Matlab.

| 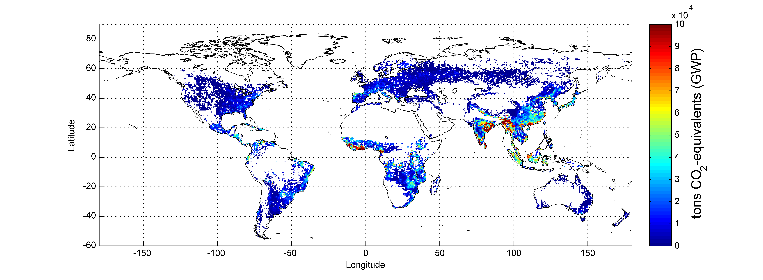  a) | 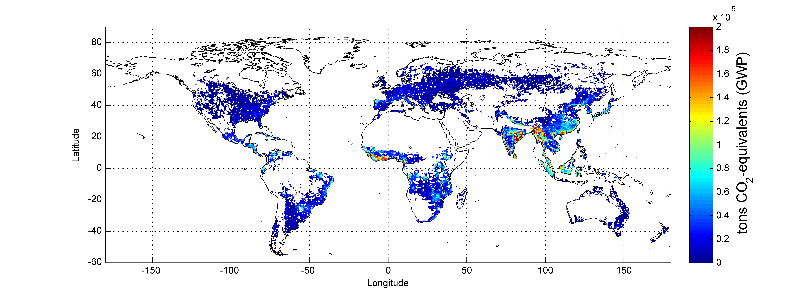  b) |
| --- | --- |
| 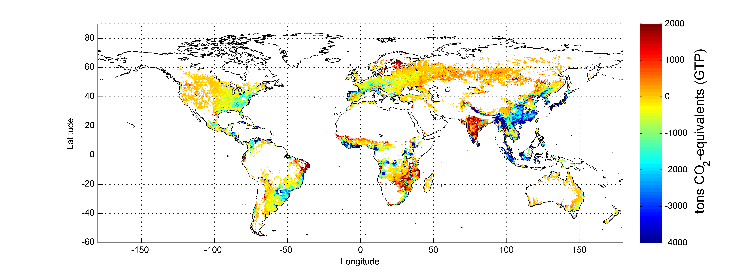  c) | 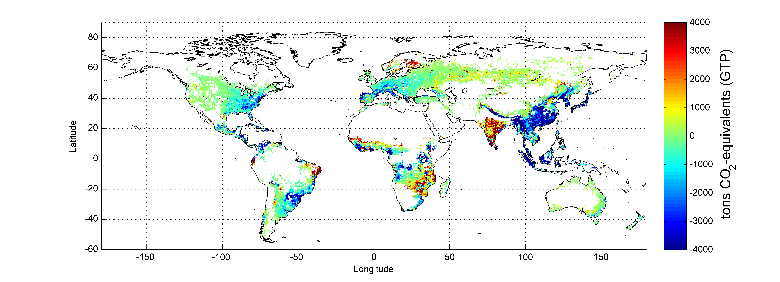  d) |
| 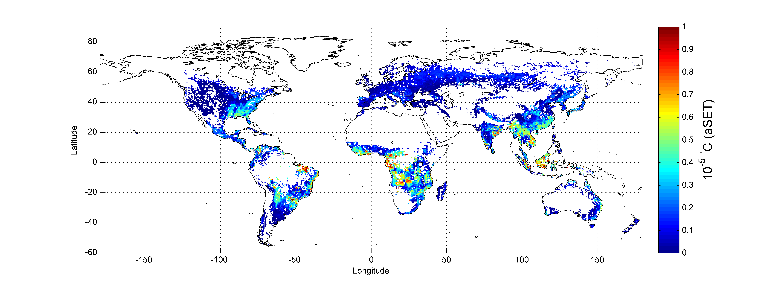  e) | 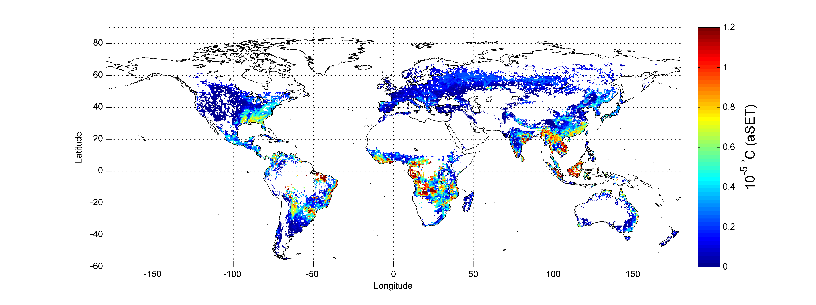  f) |

Figure S6 Climate change impacts from emissions in 2015 computed using GWP (a, b), or GTP (c, d), and from the entire emission scenario computed with aSET (e. f). Results are for the cases with 100% (a, c, e) and 0% (b, d, f) residue extraction rates. Maps are generated using the software Matlab.

Table S3 Emission flows (in 2015, maximum emission rates, and cumulative emissions over until 2100) and results aggregated at a country level for 0%, 50% and 100% residue extraction rates.

| Country | CO_2_ from forest bioenergy (2015-2100) | | | GWP 100 (Mtons CO_2_-eq.) | | | GTP 85 (ktons CO_2_-eq.) | | | aSET (10^-3^ °C) | | |
| --- | --- | --- | --- | --- | --- | --- | --- | --- | --- | --- | --- | --- |
|  | 2015  (Mton yr^-1^) | Max rate  (Mton yr^-1^) | Cumulative  (Gton) | 50% | No res | All res | 50% | No res | All res | 50% | No res | All res |
| Afghanistan | 0.02 | 0.11 | 0.01 | 0.0 | 0.0 | 0.0 | -0.8 | -0.7 | -1.0 | 0.13 | 0.11 | 0.17 |
| Albania | 3.03 | 5.84 | 0.37 | 1.6 | 1.3 | 2.1 | 237.7 | 186.2 | 358.0 | 0.04 | 0.03 | 0.05 |
| Algeria | 1.23 | 2.36 | 0.19 | 0.5 | 0.4 | 0.7 | -46.5 | -39.8 | -59.7 | 0.09 | 0.07 | 0.11 |
| Andorra | 3.61 | 4.69 | 0.34 | 1.7 | 1.4 | 2.2 | -47.1 | -44.3 | -51.5 | 0.00 | 0.00 | 0.00 |
| Angola | 0.08 | 0.10 | 0.01 | 0.0 | 0.0 | 0.0 | -4.0 | -3.4 | -5.2 | 7.02 | 5.85 | 9.49 |
| Antigua and Barbuda | 41.19 | 378.45 | 18.23 | 19.4 | 16.1 | 26.4 | 3.2 | -89.8 | 229.1 | 3.00 | 2.54 | 3.92 |
| Argentina | 31.87 | 165.53 | 11.29 | 14.1 | 11.9 | 18.3 | -621.8 | -561.2 | -724.5 | 0.02 | 0.01 | 0.02 |
| Armenia | 0.22 | 0.86 | 0.05 | 0.1 | 0.1 | 0.1 | -4.3 | -3.9 | -5.1 | 4.94 | 4.18 | 6.43 |
| Australia | 22.15 | 273.07 | 11.21 | 9.7 | 8.2 | 12.5 | -97.4 | -125.1 | -17.7 | 0.09 | 0.08 | 0.12 |
| Austria | 4.60 | 5.08 | 0.41 | 2.1 | 1.8 | 2.6 | -48.4 | -48.2 | -46.1 | 0.03 | 0.02 | 0.03 |
| Azerbaijan | 0.49 | 1.47 | 0.10 | 0.2 | 0.2 | 0.3 | 4.6 | 2.9 | 8.1 | 0.02 | 0.01 | 0.02 |
| Bahamas | 0.27 | 0.96 | 0.06 | 0.1 | 0.1 | 0.2 | -9.0 | -7.7 | -11.4 | 0.16 | 0.13 | 0.22 |
| Bangladesh | 8.50 | 8.51 | 0.60 | 4.0 | 3.3 | 5.6 | -169.4 | -147.8 | -213.3 | 0.45 | 0.39 | 0.57 |
| Barbados | 6.21 | 25.39 | 1.80 | 2.8 | 2.4 | 3.5 | -113.1 | -102.6 | -132.7 | 0.02 | 0.02 | 0.03 |
| Belarus | 0.91 | 1.16 | 0.09 | 0.4 | 0.3 | 0.5 | -31.3 | -27.2 | -38.8 | 0.06 | 0.05 | 0.08 |
| Belgium | 0.32 | 3.59 | 0.24 | 0.1 | 0.1 | 0.2 | -10.8 | -9.3 | -13.8 | 0.24 | 0.20 | 0.32 |
| Belize | 7.74 | 12.40 | 0.71 | 3.7 | 3.1 | 5.1 | 113.6 | 72.3 | 201.5 | 0.08 | 0.07 | 0.11 |
| Benin | 4.69 | 4.90 | 0.37 | 2.0 | 1.6 | 2.6 | -161.0 | -136.0 | -207.2 | 2.78 | 2.31 | 3.74 |
| Bhutan | 13.41 | 150.25 | 8.87 | 6.0 | 5.0 | 8.0 | -307.6 | -269.3 | -378.7 | 0.15 | 0.13 | 0.19 |
| Bolivia | 1.45 | 8.83 | 0.58 | 0.6 | 0.5 | 0.8 | -55.9 | -48.2 | -70.6 | 0.85 | 0.72 | 1.12 |
| Bosnia and Herzegovina | 3.55 | 41.89 | 1.55 | 1.9 | 1.6 | 2.4 | 378.6 | 298.2 | 545.0 | 17.24 | 14.56 | 22.24 |
| Botswana | 162.32 | 958.88 | 46.31 | 73.7 | 62.1 | 95.2 | -986.2 | -1092.8 | -727.3 | 0.04 | 0.04 | 0.06 |
| Brazil | 0.57 | 2.41 | 0.14 | 0.3 | 0.2 | 0.4 | -18.3 | -15.3 | -23.9 | 0.22 | 0.19 | 0.28 |
| Brunei Darussalam | 2.07 | 12.49 | 0.68 | 0.9 | 0.8 | 1.2 | -49.3 | -43.7 | -59.6 | 0.18 | 0.15 | 0.24 |
| Bulgaria | 7.70 | 9.22 | 0.62 | 3.8 | 3.1 | 5.2 | 324.9 | 239.7 | 503.1 | 0.01 | 0.01 | 0.02 |
| Burkina Faso | 0.39 | 0.74 | 0.03 | 0.2 | 0.2 | 0.2 | -7.4 | -6.7 | -8.8 | 1.27 | 1.05 | 1.68 |
| Burundi | 11.99 | 69.46 | 3.40 | 5.5 | 4.6 | 7.4 | -183.7 | -169.9 | -208.9 | 2.24 | 1.88 | 2.90 |
| Cambodia | 28.95 | 124.21 | 6.26 | 13.2 | 11.0 | 17.2 | -825.8 | -711.8 | -1037.6 | 2.60 | 2.24 | 3.30 |
| Cameroon | 12.82 | 142.55 | 7.52 | 5.9 | 5.0 | 7.4 | -11.6 | -26.2 | 19.1 | 2.46 | 2.08 | 3.18 |
| Canada | 5.99 | 135.36 | 4.36 | 2.7 | 2.3 | 3.5 | -91.4 | -84.6 | -103.5 | 0.17 | 0.14 | 0.25 |
| Canarias | 4.58 | 8.74 | 0.62 | 2.3 | 1.9 | 3.3 | 193.5 | 138.7 | 318.0 | 0.26 | 0.22 | 0.34 |
| Central African Rep. | 6.53 | 14.70 | 1.08 | 2.9 | 2.5 | 3.8 | -124.0 | -111.7 | -145.9 | 14.56 | 12.30 | 19.17 |
| Chad | 411.32 | 821.31 | 53.95 | 179.7 | 151.2 | 237.5 | -10913.4 | -9465.1 | -13728.3 | 2.39 | 1.98 | 3.16 |
| Chile | 24.70 | 132.15 | 6.49 | 11.2 | 9.3 | 14.8 | -732.6 | -626.6 | -930.2 | 0.00 | 0.00 | 0.00 |
| China | 0.13 | 0.16 | 0.01 | 0.1 | 0.1 | 0.1 | -3.7 | -3.2 | -4.6 | 1.85 | 1.56 | 2.40 |
| Colombia | 5.59 | 102.31 | 3.38 | 2.6 | 2.2 | 3.4 | -54.7 | -54.3 | -55.0 | 6.04 | 5.10 | 7.79 |
| Comoros | 75.85 | 337.62 | 21.47 | 34.0 | 28.7 | 44.0 | -1894.4 | -1661.2 | -2326.7 | 0.12 | 0.10 | 0.16 |
| Congo | 4.04 | 6.54 | 0.50 | 1.8 | 1.5 | 2.5 | -129.6 | -108.9 | -168.9 | 1.59 | 1.34 | 2.05 |
| Congo DRC | 48.30 | 86.48 | 5.65 | 22.3 | 18.8 | 28.9 | -550.8 | -530.5 | -588.9 | 0.16 | 0.14 | 0.20 |
| Costa Rica | 1.56 | 9.48 | 0.51 | 0.7 | 0.6 | 0.8 | -61.4 | -52.9 | -77.3 | 0.19 | 0.16 | 0.25 |
| Côte d'Ivoire | 7.33 | 10.84 | 0.83 | 3.2 | 2.7 | 4.3 | -238.9 | -204.7 | -305.0 | 0.00 | 0.00 | 0.00 |
| Croatia | 0.00 | 0.00 | 0.00 | 0.0 | 0.0 | 0.0 | 0.1 | 0.1 | 0.2 | 0.15 | 0.13 | 0.19 |
| Cuba | 2.46 | 8.46 | 0.60 | 1.1 | 0.9 | 1.4 | -86.2 | -75.0 | -106.9 | 0.02 | 0.02 | 0.03 |
| Cyprus | 0.40 | 1.06 | 0.06 | 0.2 | 0.2 | 0.2 | -0.3 | -0.8 | 0.6 | 0.11 | 0.09 | 0.15 |
| Czech Republic | 5.10 | 6.24 | 0.49 | 2.2 | 1.8 | 3.0 | -182.6 | -154.6 | -238.3 | 0.51 | 0.42 | 0.67 |
| Denmark | 11.70 | 27.58 | 1.85 | 5.5 | 4.6 | 7.3 | 227.5 | 160.7 | 363.0 | 0.06 | 0.05 | 0.08 |
| Dominica | 2.20 | 3.10 | 0.23 | 1.0 | 0.9 | 1.4 | 4.8 | -0.5 | 15.1 | 0.21 | 0.17 | 0.27 |
| Dominican Republic | 0.86 | 11.24 | 0.48 | 0.4 | 0.3 | 0.5 | -21.1 | -18.4 | -26.0 | 0.00 | 0.00 | 0.00 |
| Ecuador | 0.00 | 0.00 | 0.00 | 0.0 | 0.0 | 0.0 | 0.1 | 0.0 | 0.1 | 0.09 | 0.08 | 0.11 |
| El Salvador | 0.95 | 4.83 | 0.33 | 0.5 | 0.4 | 0.6 | 37.4 | 29.5 | 53.0 | 0.77 | 0.63 | 1.08 |
| Equatorial Guinea | 33.36 | 41.84 | 2.11 | 15.2 | 12.5 | 21.4 | -558.3 | -510.7 | -645.4 | 0.00 | 0.00 | 0.00 |
| Eritrea | 0.00 | 0.00 | 0.00 | 0.0 | 0.0 | 0.0 | 0.0 | 0.0 | 0.0 | 0.00 | 0.00 | 0.00 |
| Estonia | 0.00 | 0.00 | 0.00 | 0.0 | 0.0 | 0.0 | 0.0 | 0.0 | 0.0 | 0.34 | 0.29 | 0.43 |
| Ethiopia | 15.18 | 17.68 | 1.46 | 7.4 | 6.4 | 9.5 | 1039.2 | 849.1 | 1418.9 | 0.56 | 0.48 | 0.71 |
| Falkland Islands | 29.33 | 32.82 | 2.67 | 12.5 | 10.7 | 15.9 | -979.6 | -851.5 | -1219.5 | 0.01 | 0.00 | 0.01 |
| Faroe Islands | 0.04 | 0.29 | 0.01 | 0.0 | 0.0 | 0.0 | -1.1 | -1.0 | -1.5 | 1.84 | 1.55 | 2.40 |
| Fiji | 3.10 | 103.12 | 2.05 | 1.4 | 1.2 | 1.9 | -51.0 | -46.2 | -59.8 | 0.02 | 0.02 | 0.03 |
| Finland | 0.86 | 0.96 | 0.07 | 0.4 | 0.4 | 0.6 | 78.9 | 60.3 | 118.4 | 0.11 | 0.09 | 0.14 |
| France | 1.90 | 6.17 | 0.42 | 0.8 | 0.7 | 1.0 | -30.0 | -27.8 | -32.2 | 0.37 | 0.32 | 0.47 |
| French Guiana | 17.83 | 20.81 | 1.66 | 8.0 | 6.9 | 10.1 | -496.0 | -436.2 | -606.8 | 0.72 | 0.60 | 0.97 |
| Gabon | 28.61 | 39.07 | 2.28 | 13.4 | 11.1 | 18.0 | -466.4 | -426.4 | -544.5 | 0.13 | 0.11 | 0.17 |
| Gambia | 6.27 | 7.07 | 0.58 | 2.9 | 2.4 | 3.8 | -100.7 | -92.4 | -114.8 | 0.00 | 0.00 | 0.00 |
| Georgia | 0.07 | 0.18 | 0.01 | 0.0 | 0.0 | 0.0 | -2.1 | -1.8 | -2.7 | 0.12 | 0.10 | 0.16 |
| Germany | 2.79 | 6.94 | 0.46 | 1.2 | 1.0 | 1.6 | -76.5 | -65.8 | -96.3 | 0.77 | 0.64 | 1.03 |
| Ghana | 23.32 | 41.10 | 2.70 | 11.0 | 9.1 | 14.8 | -118.3 | -140.6 | -68.3 | 0.02 | 0.02 | 0.03 |
| Greece | 0.91 | 1.23 | 0.06 | 0.5 | 0.4 | 0.6 | 27.5 | 19.1 | 46.0 | 0.41 | 0.34 | 0.54 |
| Grenada | 1.23 | 22.22 | 1.18 | 0.6 | 0.5 | 0.8 | -34.2 | -29.4 | -43.5 | 0.01 | 0.01 | 0.02 |
| Guadeloupe | 0.60 | 0.82 | 0.06 | 0.3 | 0.2 | 0.4 | -23.3 | -19.6 | -30.3 | 0.33 | 0.27 | 0.43 |
| Guatemala | 11.21 | 18.22 | 1.28 | 5.1 | 4.2 | 6.7 | -312.6 | -270.9 | -392.3 | 0.14 | 0.12 | 0.18 |
| Guinea | 2.59 | 7.94 | 0.56 | 1.2 | 1.0 | 1.5 | -25.9 | -25.0 | -26.7 | 7.97 | 6.64 | 10.64 |
| Guinea-Bissau | 413.28 | 431.34 | 31.65 | 192.3 | 159.7 | 257.7 | 1251.7 | 136.3 | 3756.6 | 9.74 | 8.03 | 13.03 |
| Guyana | 168.69 | 533.38 | 33.90 | 77.4 | 63.6 | 103.8 | -4933.3 | -4196.1 | -6347.1 | 0.06 | 0.05 | 0.08 |
| Haiti | 2.32 | 3.12 | 0.23 | 1.2 | 1.0 | 1.6 | 163.7 | 126.0 | 253.4 | 0.01 | 0.01 | 0.01 |
| Honduras | 0.36 | 0.42 | 0.03 | 0.2 | 0.1 | 0.2 | 13.4 | 10.1 | 20.6 | 0.02 | 0.02 | 0.03 |
| Hungary | 0.50 | 1.22 | 0.07 | 0.2 | 0.2 | 0.3 | -16.5 | -14.3 | -20.7 | 0.01 | 0.01 | 0.01 |
| India | 0.31 | 0.48 | 0.03 | 0.2 | 0.1 | 0.2 | 29.5 | 23.1 | 43.2 | 0.33 | 0.28 | 0.42 |
| Indonesia | 17.19 | 18.52 | 1.52 | 7.7 | 6.5 | 9.8 | -316.9 | -288.6 | -366.0 | 0.05 | 0.04 | 0.06 |
| Iran | 1.08 | 2.91 | 0.18 | 0.5 | 0.4 | 0.6 | -43.0 | -36.4 | -55.2 | 1.05 | 0.89 | 1.34 |
| Iraq | 45.94 | 63.88 | 5.17 | 18.7 | 15.9 | 24.0 | -1716.8 | -1468.5 | -2179.4 | 0.00 | 0.00 | 0.00 |
| Ireland | 0.06 | 0.07 | 0.01 | 0.0 | 0.0 | 0.0 | 6.4 | 4.9 | 10.1 | 0.35 | 0.30 | 0.45 |
| Isle of Man | 2.18 | 16.74 | 0.83 | 1.1 | 1.0 | 1.5 | 236.0 | 192.8 | 328.2 | 0.40 | 0.34 | 0.52 |
| Israel | 11.81 | 21.00 | 1.10 | 5.4 | 4.6 | 6.9 | 18.7 | -7.7 | 76.3 | 0.02 | 0.02 | 0.03 |
| Italy | 0.22 | 1.24 | 0.06 | 0.1 | 0.1 | 0.1 | 5.5 | 4.3 | 8.5 | 1.92 | 1.59 | 2.59 |
| Jamaica | 16.69 | 107.12 | 5.44 | 7.5 | 6.2 | 10.2 | -515.9 | -440.2 | -667.4 | 0.13 | 0.11 | 0.16 |
| Japan | 1.95 | 7.01 | 0.51 | 0.9 | 0.8 | 1.1 | 7.0 | 3.3 | 14.0 | 0.00 | 0.00 | 0.00 |
| Jordan | 0.16 | 0.19 | 0.01 | 0.1 | 0.1 | 0.1 | 5.8 | 4.3 | 9.5 | 0.01 | 0.00 | 0.01 |
| Kazakhstan | 0.26 | 0.30 | 0.02 | 0.1 | 0.1 | 0.1 | -7.5 | -6.5 | -9.4 | 0.59 | 0.49 | 0.78 |
| Kenya | 13.46 | 32.13 | 1.95 | 6.2 | 5.1 | 8.2 | -365.8 | -314.7 | -460.7 | 0.00 | 0.00 | 0.00 |
| Kuwait | 0.04 | 0.05 | 0.00 | 0.0 | 0.0 | 0.0 | 3.4 | 2.7 | 5.0 | 0.00 | 0.00 | 0.00 |
| Kyrgyzstan | 0.02 | 0.03 | 0.00 | 0.0 | 0.0 | 0.0 | 2.1 | 1.7 | 2.9 | 0.10 | 0.09 | 0.13 |
| Laos | 1.97 | 5.53 | 0.41 | 0.9 | 0.8 | 1.1 | -16.1 | -15.9 | -16.3 | 0.00 | 0.00 | 0.00 |
| Latvia | 0.15 | 0.16 | 0.01 | 0.1 | 0.1 | 0.1 | -5.1 | -4.5 | -6.4 | 0.39 | 0.33 | 0.51 |
| Lebanon | 5.24 | 21.32 | 1.06 | 2.4 | 2.0 | 3.2 | -29.8 | -33.8 | -19.9 | 0.16 | 0.14 | 0.21 |
| Lesotho | 5.39 | 8.77 | 0.50 | 2.6 | 2.2 | 3.4 | 98.3 | 68.9 | 156.8 | 1.87 | 1.55 | 2.48 |
| Liberia | 42.92 | 106.23 | 6.14 | 19.0 | 15.7 | 25.1 | -1517.3 | -1277.9 | -1961.8 | 0.28 | 0.23 | 0.40 |
| Libya | 11.59 | 14.40 | 1.07 | 5.7 | 4.7 | 8.1 | 356.3 | 248.3 | 602.1 | 0.00 | 0.00 | 0.00 |
| Liechtenstein | 0.01 | 0.07 | 0.00 | 0.0 | 0.0 | 0.0 | 3.6 | 2.7 | 5.9 | 2.74 | 2.28 | 3.72 |
| Lithuania | 36.26 | 148.02 | 9.47 | 16.9 | 14.0 | 23.1 | -291.6 | -306.7 | -235.0 | 0.02 | 0.02 | 0.03 |
| Luxembourg | 0.51 | 1.14 | 0.09 | 0.2 | 0.2 | 0.3 | -3.5 | -3.6 | -3.1 | 0.21 | 0.18 | 0.26 |
| Madagascar | 0.83 | 10.32 | 0.41 | 0.4 | 0.4 | 0.6 | 109.4 | 92.5 | 143.6 | 0.02 | 0.02 | 0.03 |
| Malawi | 0.21 | 1.30 | 0.09 | 0.1 | 0.1 | 0.1 | -7.8 | -6.7 | -9.9 | 0.11 | 0.09 | 0.15 |
| Malaysia | 5.11 | 5.87 | 0.46 | 2.4 | 2.0 | 3.2 | -23.7 | -29.1 | -8.1 | 3.29 | 2.73 | 4.48 |
| Mali | 40.90 | 172.00 | 8.46 | 19.7 | 16.3 | 26.9 | 760.5 | 512.8 | 1294.4 | 3.53 | 2.90 | 4.87 |
| Mauritania | 140.12 | 194.63 | 13.99 | 64.3 | 52.6 | 89.1 | -3994.9 | -3411.1 | -5212.9 | 0.43 | 0.36 | 0.59 |
| Mauritius | 1.33 | 20.73 | 0.93 | 0.7 | 0.6 | 0.9 | 112.3 | 86.0 | 171.6 | 0.36 | 0.31 | 0.47 |
| Mayotte | 18.85 | 21.59 | 1.69 | 7.8 | 6.6 | 10.2 | -491.4 | -427.8 | -606.4 | 0.02 | 0.02 | 0.03 |
| Mexico | 0.44 | 1.24 | 0.06 | 0.2 | 0.2 | 0.3 | -10.2 | -9.0 | -12.3 | 0.14 | 0.12 | 0.18 |
| Moldova | 0.36 | 7.77 | 0.22 | 0.2 | 0.1 | 0.2 | -12.3 | -10.5 | -16.0 | 0.18 | 0.16 | 0.24 |
| Mongolia | 2.43 | 11.29 | 0.79 | 1.0 | 0.8 | 1.3 | -89.3 | -76.6 | -112.9 | 0.36 | 0.30 | 0.47 |
| Montenegro | 7.76 | 19.46 | 1.35 | 3.6 | 3.0 | 4.8 | -128.8 | -116.6 | -151.4 | 0.01 | 0.00 | 0.01 |
| Morocco | 0.03 | 0.20 | 0.01 | 0.0 | 0.0 | 0.0 | 13.3 | 10.2 | 20.8 | 0.71 | 0.60 | 0.93 |
| Mozambique | 28.85 | 38.22 | 2.22 | 13.5 | 11.3 | 17.7 | -64.0 | -105.5 | 18.7 | 0.35 | 0.29 | 0.46 |
| Myanmar | 8.98 | 19.93 | 1.31 | 3.9 | 3.3 | 5.1 | -264.7 | -228.6 | -338.1 | 0.10 | 0.09 | 0.13 |
| Namibia | 4.42 | 5.24 | 0.42 | 2.2 | 1.9 | 2.9 | 202.5 | 154.3 | 303.7 | 0.12 | 0.10 | 0.15 |
| Nepal | 4.60 | 6.55 | 0.44 | 1.9 | 1.6 | 2.6 | 35.4 | 17.9 | 83.7 | 0.00 | 0.00 | 0.00 |
| Netherlands | 0.04 | 0.08 | 0.01 | 0.0 | 0.0 | 0.0 | 4.1 | 3.2 | 5.8 | 0.21 | 0.17 | 0.27 |
| New Caledonia | 3.18 | 11.36 | 0.64 | 1.5 | 1.2 | 1.9 | -83.6 | -72.2 | -104.2 | 0.28 | 0.23 | 0.38 |
| New Zealand | 6.26 | 15.47 | 0.83 | 2.8 | 2.3 | 3.8 | -194.4 | -164.7 | -251.3 | 1.68 | 1.42 | 2.20 |
| Nicaragua | 19.25 | 89.94 | 5.88 | 8.3 | 7.0 | 10.8 | -610.7 | -528.1 | -760.7 | 0.34 | 0.29 | 0.45 |
| Niger | 0.34 | 18.54 | 0.82 | 0.2 | 0.1 | 0.2 | 15.1 | 11.3 | 23.9 | 0.33 | 0.27 | 0.44 |
| Nigeria | 14.58 | 18.63 | 0.89 | 6.5 | 5.4 | 8.6 | -498.6 | -420.9 | -643.6 | 0.52 | 0.45 | 0.66 |
| North Korea | 11.11 | 29.02 | 2.24 | 5.0 | 4.3 | 6.4 | -289.8 | -255.7 | -352.8 | 0.14 | 0.12 | 0.19 |
| Norway | 7.35 | 8.11 | 0.67 | 3.2 | 2.6 | 4.2 | -180.5 | -158.1 | -225.1 | 0.10 | 0.08 | 0.14 |
| Oman | 0.50 | 5.61 | 0.32 | 0.2 | 0.2 | 0.3 | -17.2 | -14.4 | -23.2 | 0.00 | 0.00 | 0.00 |
| Pakistan | 0.03 | 0.05 | 0.00 | 0.0 | 0.0 | 0.0 | -0.9 | -0.8 | -1.1 | 0.41 | 0.35 | 0.51 |
| Palestinian Territory | 3.81 | 22.87 | 1.45 | 1.7 | 1.5 | 2.2 | -120.1 | -104.7 | -148.6 | 16.88 | 14.57 | 21.53 |
| Panama | 88.31 | 869.57 | 41.88 | 42.0 | 36.2 | 53.7 | 3381.1 | 2733.4 | 4718.6 | 0.02 | 0.02 | 0.02 |
| Papua New Guinea | 1.01 | 1.05 | 0.05 | 0.4 | 0.4 | 0.6 | -28.3 | -24.7 | -34.9 | 0.00 | 0.00 | 0.00 |
| Paraguay | 0.00 | 0.16 | 0.01 | 0.0 | 0.0 | 0.0 | -0.1 | -0.1 | -0.1 | 0.00 | 0.00 | 0.00 |
| Peru | 0.01 | 0.01 | 0.00 | 0.0 | 0.0 | 0.0 | 0.2 | 0.1 | 0.4 | 0.11 | 0.09 | 0.15 |
| Philippines | 3.74 | 5.46 | 0.36 | 1.9 | 1.6 | 2.6 | 267.8 | 202.0 | 408.9 | 0.18 | 0.16 | 0.23 |
| Poland | 2.54 | 10.77 | 0.72 | 1.1 | 0.9 | 1.4 | -89.3 | -77.5 | -111.6 | 0.39 | 0.32 | 0.55 |
| Portugal | 17.10 | 20.66 | 1.32 | 8.2 | 6.6 | 11.5 | -390.5 | -335.7 | -504.8 | 0.13 | 0.11 | 0.17 |
| Puerto Rico | 1.70 | 7.46 | 0.54 | 0.8 | 0.7 | 1.0 | -62.0 | -53.7 | -77.2 | 0.08 | 0.07 | 0.10 |
| Qatar | 1.32 | 4.56 | 0.30 | 0.6 | 0.5 | 0.7 | -46.5 | -40.3 | -58.0 | 0.08 | 0.06 | 0.11 |
| Réunion | 1.46 | 4.34 | 0.33 | 0.7 | 0.5 | 0.9 | -49.3 | -41.1 | -64.8 | 0.11 | 0.09 | 0.15 |
| Romania | 0.96 | 4.07 | 0.22 | 0.7 | 0.5 | 0.9 | 300.2 | 230.4 | 457.2 | 1.31 | 1.10 | 1.73 |
| Russian Federation | 29.38 | 71.49 | 4.53 | 13.0 | 11.0 | 17.1 | -294.9 | -299.6 | -254.8 | 0.24 | 0.20 | 0.31 |
| Rwanda | 12.78 | 14.24 | 1.03 | 5.3 | 4.4 | 6.9 | -505.2 | -430.4 | -652.4 | 0.29 | 0.23 | 0.42 |
| Saint Kitts and Nevis | 9.41 | 14.91 | 1.01 | 4.6 | 3.7 | 6.7 | 70.9 | 31.2 | 169.2 | 0.61 | 0.51 | 0.80 |
| Saint Pierre & Miquelon | 30.37 | 34.82 | 2.86 | 13.3 | 11.2 | 17.6 | -812.3 | -708.9 | -1015.2 | 0.29 | 0.23 | 0.42 |
| Sao Tome and Principe | 14.44 | 14.94 | 1.11 | 7.0 | 5.6 | 10.3 | -43.1 | -68.1 | 26.0 | 0.02 | 0.02 | 0.03 |
| Saudi Arabia | 0.64 | 1.04 | 0.07 | 0.3 | 0.3 | 0.5 | 20.2 | 13.9 | 35.8 | 0.05 | 0.04 | 0.07 |
| Senegal | 0.22 | 2.84 | 0.15 | 0.1 | 0.1 | 0.1 | -7.5 | -6.4 | -9.5 | 0.07 | 0.06 | 0.10 |
| Serbia | 1.66 | 3.94 | 0.29 | 0.7 | 0.6 | 1.0 | -26.8 | -25.0 | -29.0 | 0.24 | 0.21 | 0.31 |
| Sierra Leone | 10.09 | 12.87 | 1.04 | 4.8 | 4.2 | 6.2 | 459.6 | 368.4 | 643.5 | 0.03 | 0.03 | 0.04 |
| Singapore | 1.56 | 1.72 | 0.14 | 0.7 | 0.6 | 0.9 | -41.8 | -37.0 | -50.2 | 0.01 | 0.01 | 0.01 |
| Slovakia | 0.32 | 0.38 | 0.03 | 0.2 | 0.1 | 0.2 | 13.1 | 10.0 | 20.0 | 0.01 | 0.01 | 0.01 |
| Slovenia | 0.02 | 0.32 | 0.01 | 0.0 | 0.0 | 0.0 | 0.9 | 0.7 | 1.3 | 3.01 | 2.51 | 4.09 |
| Solomon Islands | 64.06 | 159.05 | 9.79 | 30.6 | 25.4 | 41.6 | 684.5 | 405.4 | 1294.5 | 1.68 | 1.39 | 2.32 |
| Somalia | 56.42 | 92.41 | 6.62 | 25.8 | 21.1 | 35.6 | -1569.8 | -1347.3 | -2027.2 | 0.07 | 0.06 | 0.08 |
| South Africa | 1.29 | 3.64 | 0.27 | 0.6 | 0.5 | 0.8 | -24.7 | -22.2 | -29.3 | 0.05 | 0.04 | 0.07 |
| South Korea | 0.25 | 2.77 | 0.16 | 0.1 | 0.1 | 0.2 | -2.7 | -2.6 | -2.8 | 0.08 | 0.07 | 0.11 |
| South Sudan | 2.62 | 4.39 | 0.23 | 1.2 | 1.0 | 1.6 | -22.5 | -23.0 | -21.1 | 0.03 | 0.03 | 0.04 |
| Spain | 1.32 | 1.83 | 0.15 | 0.6 | 0.5 | 0.8 | -43.3 | -36.8 | -55.9 | 0.03 | 0.02 | 0.04 |
| Sri Lanka | 1.06 | 1.36 | 0.10 | 0.5 | 0.4 | 0.7 | 19.5 | 13.0 | 36.7 | 0.16 | 0.14 | 0.20 |
| Sudan | 7.07 | 8.89 | 0.68 | 3.2 | 2.7 | 4.1 | -163.6 | -145.0 | -197.4 | 0.00 | 0.00 | 0.00 |
| Suriname | 0.01 | 0.01 | 0.00 | 0.0 | 0.0 | 0.0 | 0.6 | 0.4 | 0.9 | 0.32 | 0.27 | 0.43 |
| Swaziland | 14.78 | 17.83 | 0.82 | 6.6 | 5.5 | 8.9 | -470.5 | -401.6 | -612.2 | 0.56 | 0.48 | 0.70 |
| Sweden | 11.75 | 31.41 | 2.33 | 5.3 | 4.5 | 6.6 | -168.3 | -156.5 | -189.2 | 0.06 | 0.05 | 0.07 |
| Switzerland | 1.86 | 2.96 | 0.19 | 0.9 | 0.7 | 1.1 | -18.9 | -19.1 | -17.5 | 12.86 | 10.97 | 16.44 |
| Syria | 126.23 | 758.34 | 50.98 | 53.4 | 45.5 | 68.4 | -3940.5 | -3422.7 | -4897.3 | 0.14 | 0.12 | 0.18 |
| Tajikistan | 2.85 | 8.49 | 0.61 | 1.1 | 1.0 | 1.5 | -115.0 | -97.0 | -151.5 | 0.00 | 0.00 | 0.00 |
| Tanzania | 0.01 | 0.03 | 0.00 | 0.0 | 0.0 | 0.0 | 1.3 | 1.0 | 2.0 | 0.01 | 0.00 | 0.01 |
| Thailand | 0.14 | 0.30 | 0.02 | 0.1 | 0.1 | 0.1 | -4.9 | -4.1 | -6.3 | 1.65 | 1.38 | 2.14 |
| Macedonia | 34.30 | 89.73 | 6.07 | 15.9 | 13.4 | 20.8 | -124.8 | -169.2 | -18.8 | 1.68 | 1.40 | 2.26 |
| Timor-Leste | 49.36 | 94.14 | 6.68 | 22.2 | 18.4 | 30.0 | -1432.5 | -1230.1 | -1833.8 | 0.03 | 0.02 | 0.04 |
| Togo | 0.43 | 1.35 | 0.07 | 0.2 | 0.2 | 0.3 | 36.5 | 27.1 | 59.9 | 3.87 | 3.25 | 5.15 |
| Trinidad and Tobago | 32.47 | 206.70 | 10.75 | 15.5 | 12.9 | 20.6 | 448.2 | 293.7 | 768.2 | 1.76 | 1.47 | 2.36 |
| Tunisia | 35.11 | 90.81 | 5.63 | 16.9 | 14.1 | 22.7 | 889.8 | 638.9 | 1433.2 | 0.13 | 0.11 | 0.17 |
| Turkey | 0.02 | 0.11 | 0.01 | 0.0 | 0.0 | 0.0 | -0.8 | -0.7 | -1.0 | 0.04 | 0.03 | 0.05 |
| Turkmenistan | 3.03 | 5.84 | 0.37 | 1.6 | 1.3 | 2.1 | 237.7 | 186.2 | 358.0 | 0.09 | 0.07 | 0.11 |
| Turks & Caicos Islands | 1.23 | 2.36 | 0.19 | 0.5 | 0.4 | 0.7 | -46.5 | -39.8 | -59.7 | 0.00 | 0.00 | 0.00 |
| Uganda | 3.61 | 4.69 | 0.34 | 1.7 | 1.4 | 2.2 | -47.1 | -44.3 | -51.5 | 7.02 | 5.85 | 9.49 |
| Ukraine | 0.08 | 0.10 | 0.01 | 0.0 | 0.0 | 0.0 | -4.0 | -3.4 | -5.2 | 3.00 | 2.54 | 3.92 |
| United Arab Emirates | 41.19 | 378.45 | 18.23 | 19.4 | 16.1 | 26.4 | 3.2 | -89.8 | 229.1 | 0.02 | 0.01 | 0.02 |
| United Kingdom | 31.87 | 165.53 | 11.29 | 14.1 | 11.9 | 18.3 | -621.8 | -561.2 | -724.5 | 4.94 | 4.18 | 6.43 |
| United States | 0.22 | 0.86 | 0.05 | 0.1 | 0.1 | 0.1 | -4.3 | -3.9 | -5.1 | 0.09 | 0.08 | 0.12 |
| Uruguay | 22.15 | 273.07 | 11.21 | 9.7 | 8.2 | 12.5 | -97.4 | -125.1 | -17.7 | 0.03 | 0.02 | 0.03 |
| Uzbekistan | 4.60 | 5.08 | 0.41 | 2.1 | 1.8 | 2.6 | -48.4 | -48.2 | -46.1 | 0.02 | 0.01 | 0.02 |
| Vanuatu | 0.49 | 1.47 | 0.10 | 0.2 | 0.2 | 0.3 | 4.6 | 2.9 | 8.1 | 0.16 | 0.13 | 0.22 |
| Venezuela | 0.27 | 0.96 | 0.06 | 0.1 | 0.1 | 0.2 | -9.0 | -7.7 | -11.4 | 0.45 | 0.39 | 0.57 |
| Vietnam | 8.50 | 8.51 | 0.60 | 4.0 | 3.3 | 5.6 | -169.4 | -147.8 | -213.3 | 0.02 | 0.02 | 0.03 |
| Yemen | 6.21 | 25.39 | 1.80 | 2.8 | 2.4 | 3.5 | -113.1 | -102.6 | -132.7 | 0.06 | 0.05 | 0.08 |
| Zambia | 0.91 | 1.16 | 0.09 | 0.4 | 0.3 | 0.5 | -31.3 | -27.2 | -38.8 | 0.24 | 0.20 | 0.32 |
| Zimbabwe | 0.32 | 3.59 | 0.24 | 0.1 | 0.1 | 0.2 | -10.8 | -9.3 | -13.8 | 0.08 | 0.07 | 0.11 |
| Total | 2986 | 9493 | 553 | 1357 | 1134 | 1800 | -36813 | -34868 | -39350 | 173 | 146 | 227 |

**References**

1 Luyssaert, S. *et al.* Land management and land-cover change have impacts of similar magnitude on surface temperature. *Nature Clim. Change* **4**, 389-393 (2014).

2 Roberts, T. L. D. & Froese, R. E. Understanding Recovery and Sustainability of Forest Residue Harvest. 41 (Michigan Technological University, Houghton, MI 49931, U.S.A., 2011).

3 Joos, F. *et al.* Carbon dioxide and climate impulse response functions for the computation of greenhouse gas metrics: a multi-model analysis. *Atmos. Chem. Phys.* **13**, 2793-2825 (2013).
